# Supplementary material for: Understanding and tailoring ligand interactions in the self-assembly of branched colloidal nanocrystals into planar superlattices
Source: Nat Commun. 2018 Mar 20;9:1141. doi: 10.1038/s41467-018-03550-z (PMC5861251; doi:10.1038/s41467-018-03550-z)
Supplement: Supplementary file 1 — Supplementary Information(PDF 66729 kb) [file 41467_2018_3550_MOESM1_ESM.pdf]

Understanding and Tailoring Ligand Interactions in the Self-Assembly of Branched Colloidal Nanocrystals into Planar Superlattices

Castelli et al.

Supplementary Note 1: Synthesis of octapods

Chemicals. Copper(II) acetylacetonate ($Cu(acac)_2$, 99.99%), cadmium chloride ($CdCl_2$, 99.99%), 1-hexanethiol (HT, 95%), 1- dodecanethiol (DDT, ≥98%), 1-octadecanethiol (ODT, 98%), oleylamine (OLAM, 70%), and octadecylphosphonic acid (ODPA, 97%) were purchased from Sigma-Aldrich. Selenium powder (Se, 99.99%), sulfur powder (S, 99%), cadmium oxide powder (CdO, 99.999%), tri-n-octylphosphine oxide (TOPO, 99%), and tri-n-octylphosphine (TOP,97%) were purchased from Strem Chemicals. Hexylphosphonic acid (HPA) was purchased from Polycarbon Industries. All chemicals were used as received and all the syntheses were carried out using a standard Schlenk line.

Preformed $Cu_{2-x}Se$ seeds[1]. In a three-neck flask 1 mmol of $Cu(acac)_2$ (262 mg) was dissolved in 9.5 ml of OLAM and 3 ml of DDT and degassed for 1h at 60°C under stirring. Then, the temperature was increased to 220°C under N_2 flow. Once the solution became orange-brown, 1 ml of a previously prepared solution of Se (1 M in OLAM-DDT mixture 50-50 % vol.) and 1.5 ml of degassed DDT were injected in the flask. After 4 min, the flask was cooled to room temperature and the solution was transferred to a N_2 filled glovebox to proceed with several washing cycles via precipitation with methanol and re-dissolution in toluene to eliminate the excess of ligands from the synthesis.

CdSe/CdS octapods[2, 3]. In a three-neck flask 60 mg of CdO, 6 mg of $CdCl_2$, 3 g of TOPO, 290 mg of ODPA, and 80 mg of HPA were degassed for 1h at 130°C under stirring. The solution was heated up to 350°C under N_2 flow. When the solution became transparent (≈ 260°C) 2.5 ml of anhydrous TOP were injected in the flask. Once the temperature reached 350°C, the $Cu_{2-x}Se$ seeds previously prepared, 500 μl of TOP, and 620 μl of S precursor (32 mg/ml solution in TOP) were quickly injected in the mixture. The reaction was run for 10 min before cooling it down to room temperature. When the temperature dropped below 100°C, 3 ml of anhydrous toluene were added to the resulting product. The resulting solution was then washed twice using a mixture of ethanol and methanol and centrifuged, to remove the excess of free-ligands in the suspension that can induce particle aggregation in solution. The pod size was tuned by using different amounts of $Cu_{2-x}Se$ seeds: 1.8 nmol, 1.1 nmol, 0.3 nmol, and 0.2 nmol for octapods with L/D of 3.0, 3.5, 5.0, and 6.0, respectively. All the samples were stored under atmospheric conditions and the assembly experiments were performed under open-air conditions in a cleanroom.

Supplementary Note 2: Details of the as-synthesized octapod-shaped nanocrystals

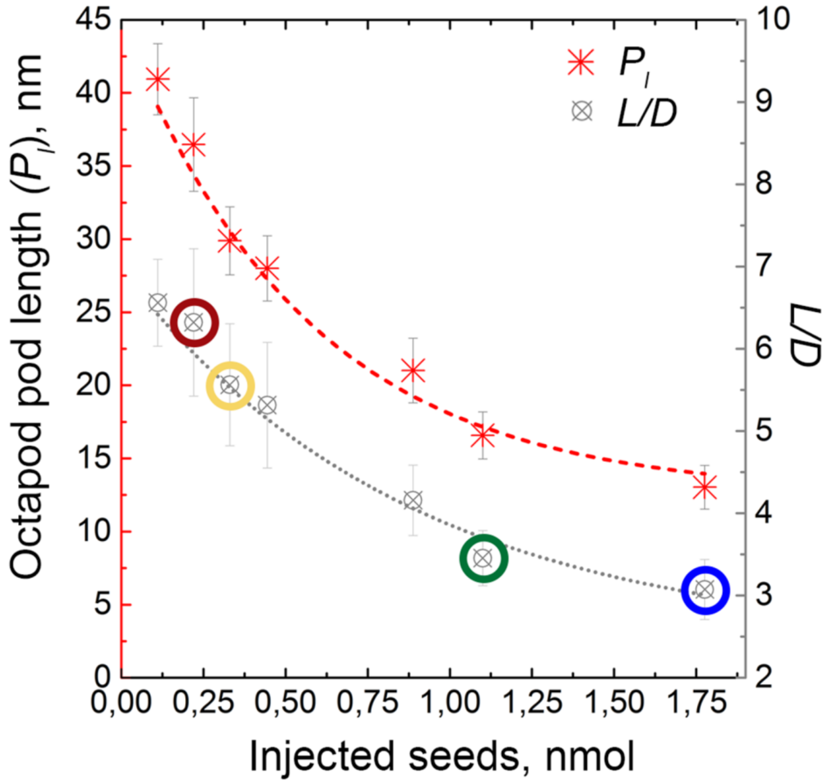

Supplementary Figure 1. Average octapod pod length (P_l) and L/D aspect ratio as a function of amount of injected seeds of $Cu_{2-x}Se$ nanocrystals. The color-framed points in the L/D curve correspond to the selected octapod batches. Standard deviations are represented by error bars and it was evaluated from from octapods obtained from four replicas of the described synthesis protocol.

The resulting values of the geometric parameters as calculated via TEM analysis for the selected octapod sizes are shown in Supplementary Table 1. Data are presented as mean ± standard deviation.

***L*, nm**	***D*, nm**	**P_l, nm**	**L/D**
86 ± 6	14 ± 2	37 ± 3	6.0
73 ± 5	15 ± 2	30 ± 2	5.0
46 ± 3	13.4 ± 1.4	16.6 ± 1.6	3.5
39 ± 3	12.8 ± 1.5	13.0 ± 1.5	3.0

Supplementary Table 1. Octapod dimensions for the selected octapod batches as evaluated through TEM analysis. Note the use of color for the L/D values, which we will use throughout to indicate a specific sample or batch of octapods. Data are presented as mean ± standard deviation.

As can be appreciated in Supplementary Fig. 1b, the pod length (P_l) scales exponentially with the seed amount below a threshold value of approx. 2 nmol. In the range of 1.75 nmol to

0.20 nmol octapods are the main product of the synthesis; starting from an L/D of 3.0. Reducing the injected seed amount to values as low as 0.20 nmol, enabled us to synthesize octapods with a pod length that reaches a maximum of 40 nm, or, equivalently, octapods with an L/D of 6.0. Below this concentration we observed a significant amount of nanorods in the final product of the synthesis (Supplementary Fig. 2b). At a $Cu_{2-x}Se$ seed content exceeding 2 nmols, the octapod shape became compromised and we observed mainly structures with a tetrahedral symmetry (Supplementary Fig. 2c). Supplementary Fig. 2a shows the nanocrystals that form the core of the octapods. Such particles are obtained eliminating the S precursor from the synthesis of octapods, so that the only reaction taking place is the cation exchange of the $Cu_{2-x}Se$ seeds.

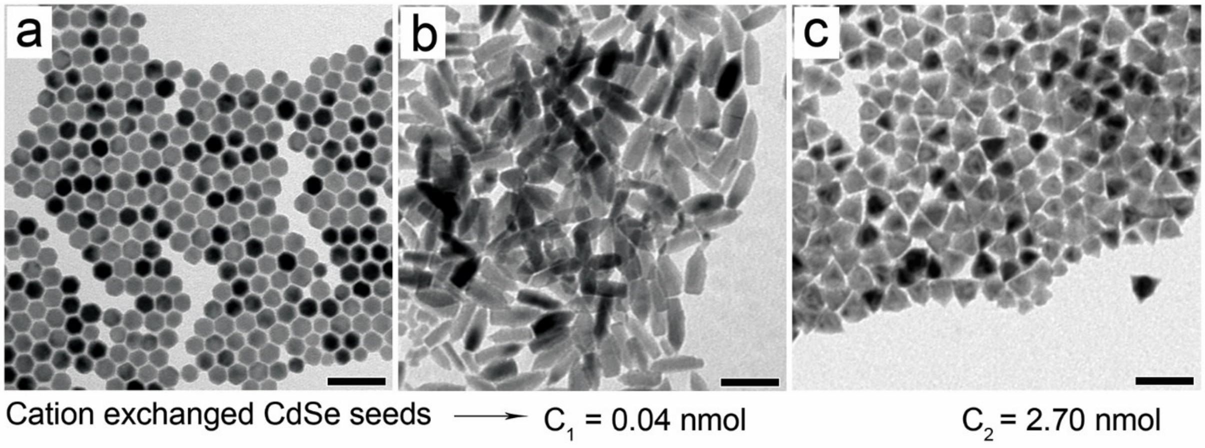

Supplementary Figure 2. TEM images of (a) Cd-cation exchanged $Cu_{2-x}Se$ nanocrystals used for the octapod syntheses. (b) Nanocrystals obtained by using a reduced amount of seeds, concentration C_1, that leads to very few particles with the octapod shape. (c) Nanocrystals synthesized with an excess of seeds, concentration C_2. The main product of this synthesis are tetrahedral particles with an edge length of approximately 20 nm. Scale bars: 50 nm.

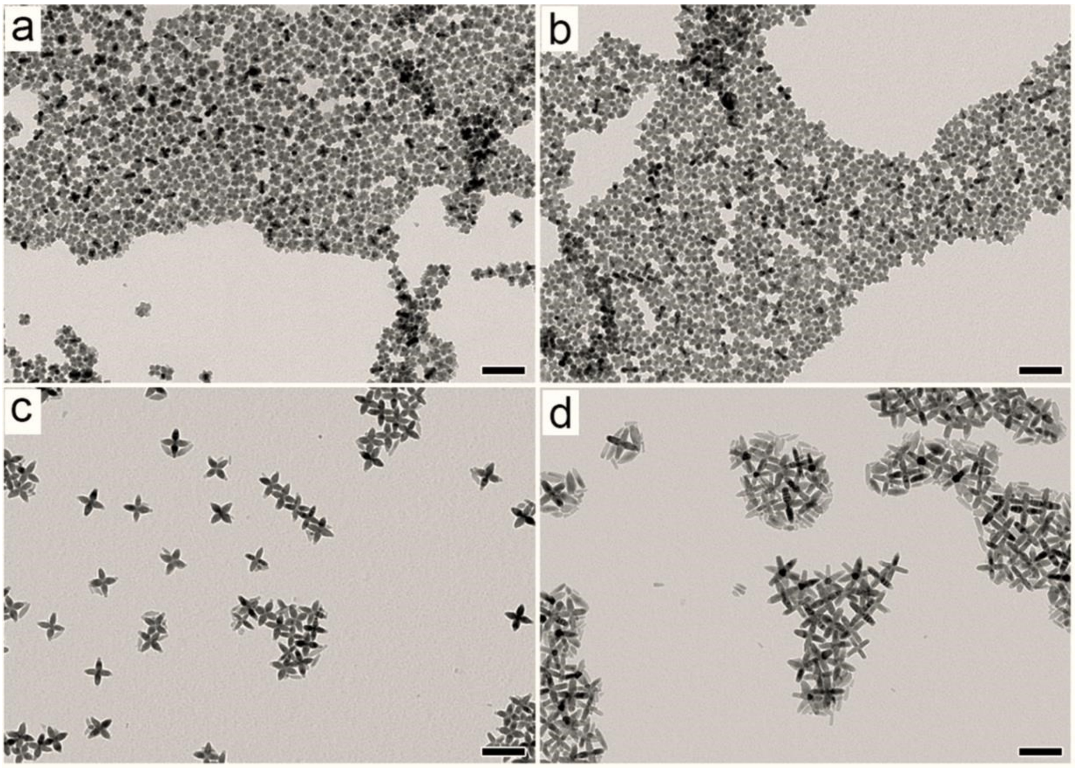

Supplementary Figure 3. Collection of low magnification TEM images of the selected octapod batches described in the main text following their aspect ratios: (a) $L/D = 3.0$; (b) $L/D = 3.5$; (c) $L/D = 5.0$; and (d) $L/D = 6.0$. Scale bars: 100 nm.

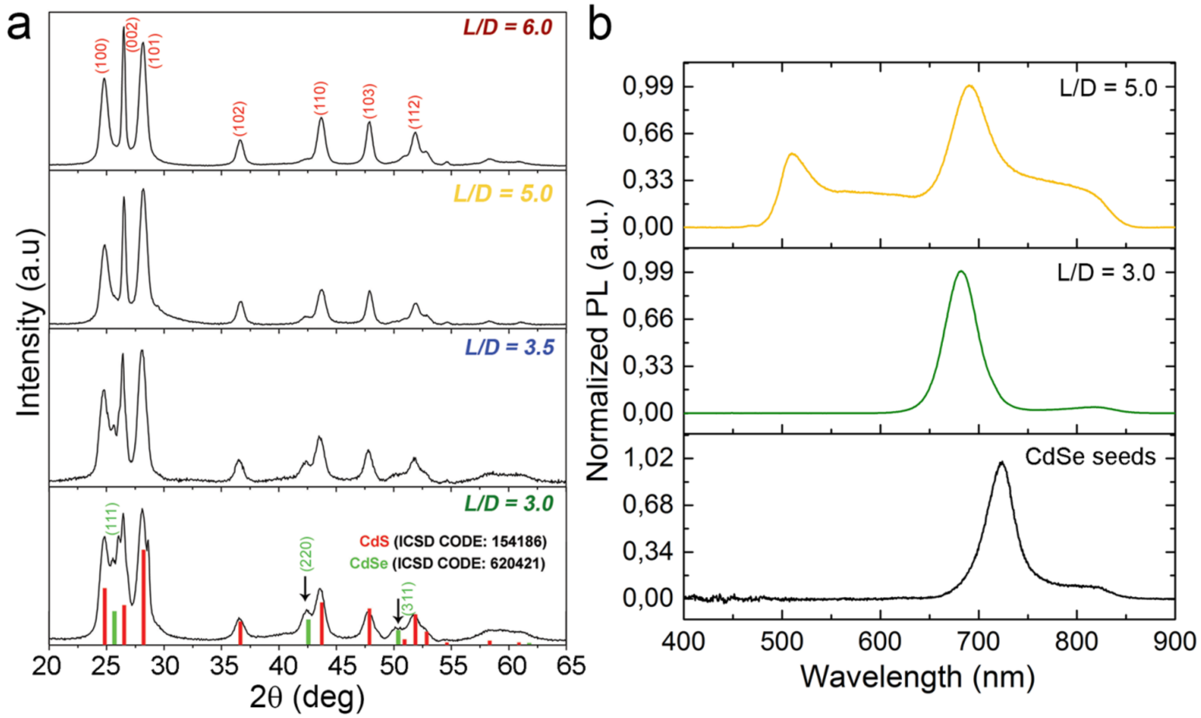

Supplementary Figure 4. (a) XRD patterns collected from the selected baches of octapod-shaped nanocrystals. (b) Photoluminisce spectra from octapods with two different sizes compared to the spectrum collected from the CdSe particles that form their cores.

As can be seen in Fig. Supplementary 4a, which evidences the growth of the CdS pods on the {111} facets of the CdSe seeds[2, 4], the sphalerite structure of the core remains clearly visible in octapods with a low L/D, while it becomes strongly shadowed by the wurtzite CdS pods that act as a large shell in particles with a higher L/D. An interesting feature of the octapods is the change in their photoluminescence, as a function of their pod length (Supplementary Fig. 4b). This change consists mainly of the appearance of the pods' CdS emission (peak at ca. 510 nm) and a broad emission at long wavelengths (from ca. 620 nm to 870 nm) of the CdSe peak for particles with longer pods ($L/D = 5.0$). Octapods with lower L/D exhibit solely a blue-shifted emission (690 nm) related to the exposed CdSe core (725 nm). Such a shift is also observed at higher L/D and it is attributed to the compressive stress induced by the CdSe-CdS lattice mismatch. In addition, the growth of longer pods favours the formation of more chemical defects at the surface (i.e., sulphur vacancies and oxygen impurities), which can act as recombination points[5] generating the observed broader emission with a peak at 820 nm for octapods with higher L/D. Further work will be conducted to explore in detail the observed changes on octapod emission.

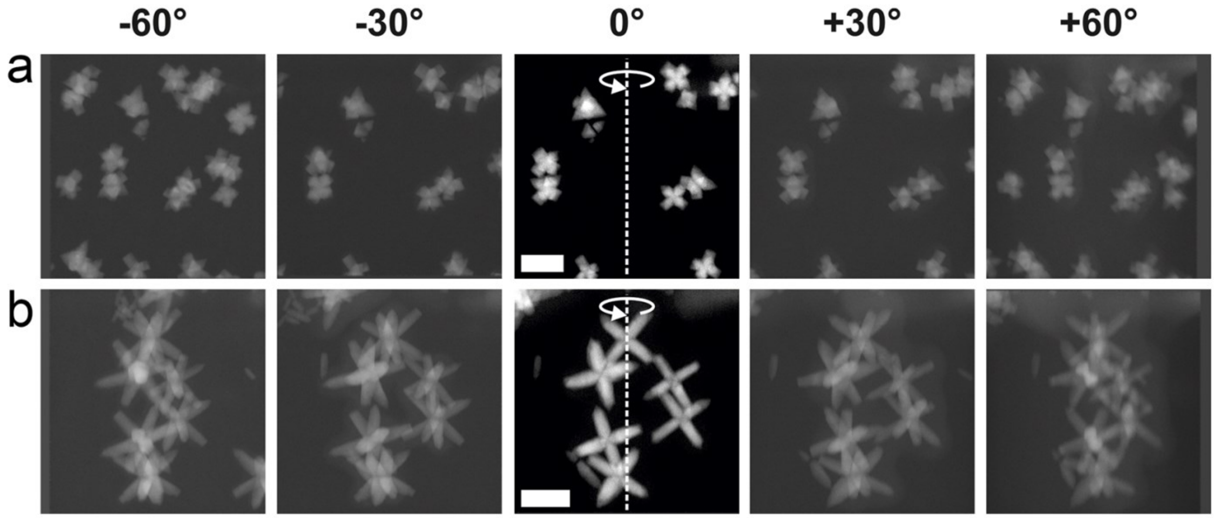

Supplementary Figure 5. HAADF-STEM images extracted from tilted series used for the reconstructions shown in movies S1 and S2 and acquired from octapods with two different L/D: (a) 3.0 and (b) 6.0. Scale bars: 50 nm.

Supplementary Note 3: Self-assembled planar superlattices from as-synthesized octapods

The interfacial assembly approach shown in Supplementary Fig. 6 constrains the octapods to move in a 2D plane, thus facilitating the analysis of octapod-octapod interactions through their ligands. It also helps to exclude by-products of the synthesis. All the suspensions were repeatedly-washed prior to being cast at the liquid interface, since the excess of organics can be a driving force for the aggregation of particles.

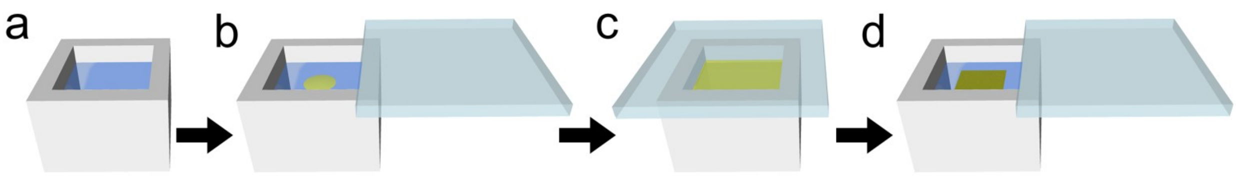

Supplementary Figure 6. Schematic illustrating the liquid-liquid interface self-assembly technique employed in this work[6]. A Teflon well with a volume capacity of 4.5 ml was filled with 2 ml of diethylene glycol (a). Next, an aliquot of 20 µl of the octapod suspension in hexane was drop cast on the top of the diethylene glycol (b). The well was kept closed for 10 min by placing a glass microslide on top of it (c). After this period, the glass microslide was removed to allow for complete evaporation of the hexane layer (d). Finally, the yellow-colored floating membrane formed at the interface with the diethylene glycol was collected on the desired substrates.

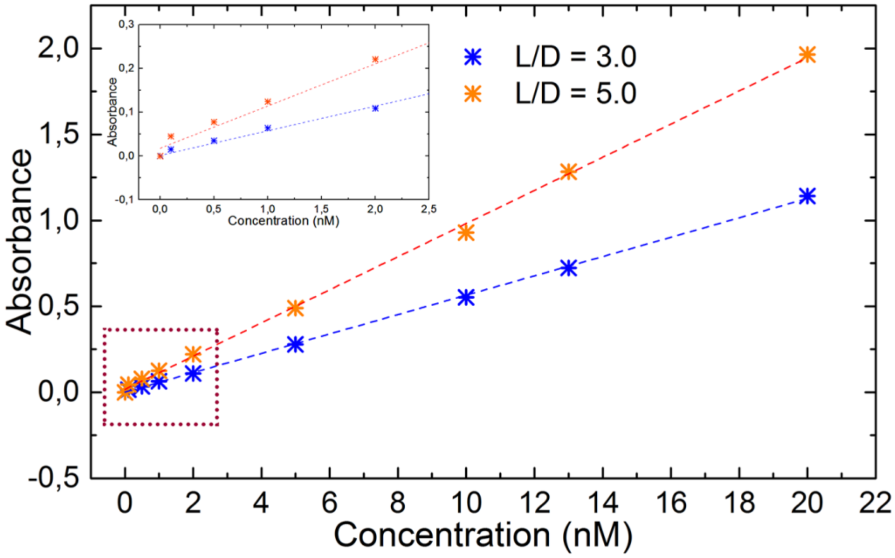

Supplementary Figure 7. Absorbance of octapod solutions, collected at an excitation wavelenght of 450 nm, as a function of the particle concentration for two different aspect ratios, L/D of 3.0 and 5.0. A zoom of the framed region is shown in the inset (for octapod concentrations below 2 nM).

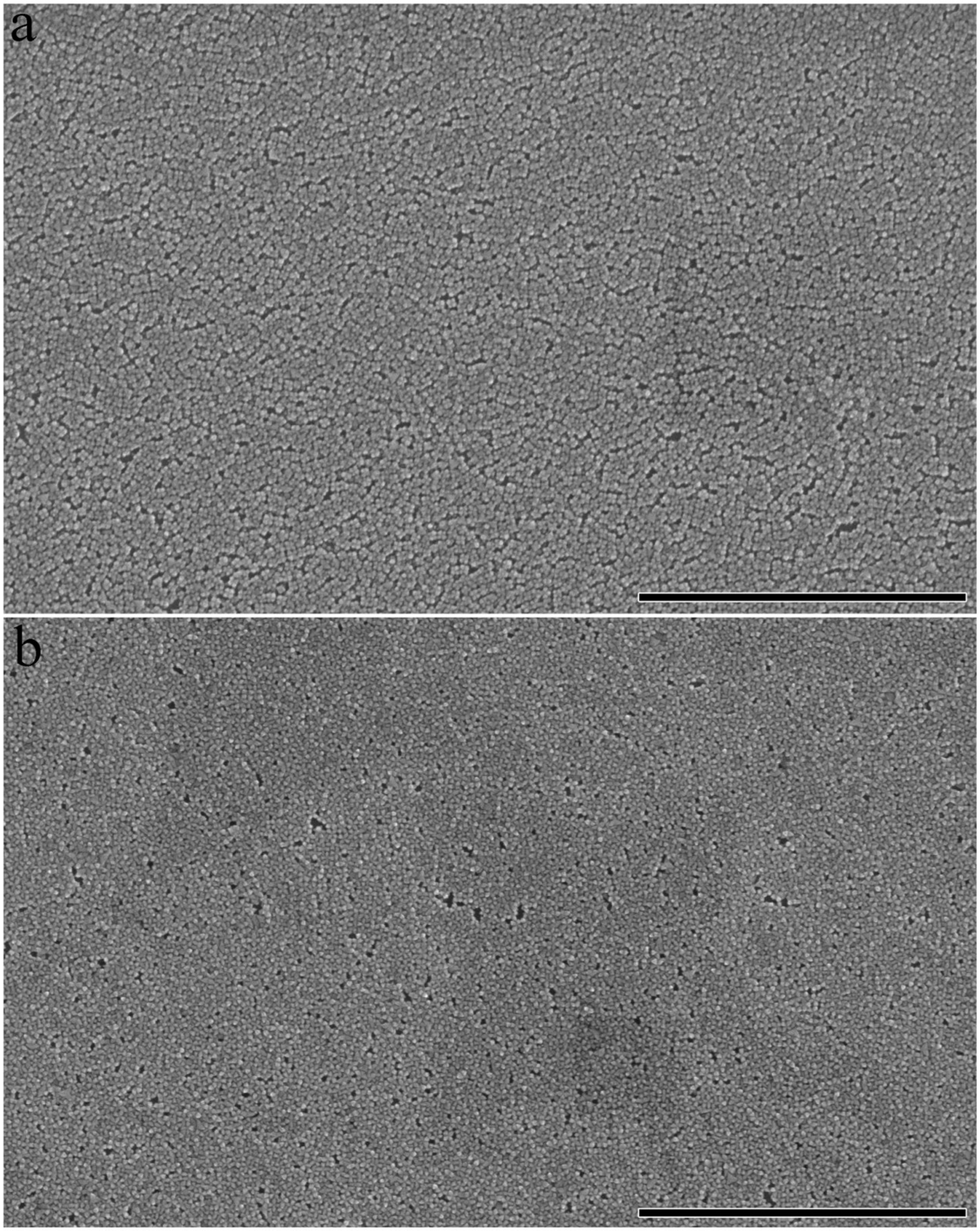

Supplementary Figure 8. Low-mag SEM view of the self-assembled planar superlattices formed by octapods with L/D of 3.0 (a) and 3.5 (b) extended over large regions. Scale bars: 1 μm.

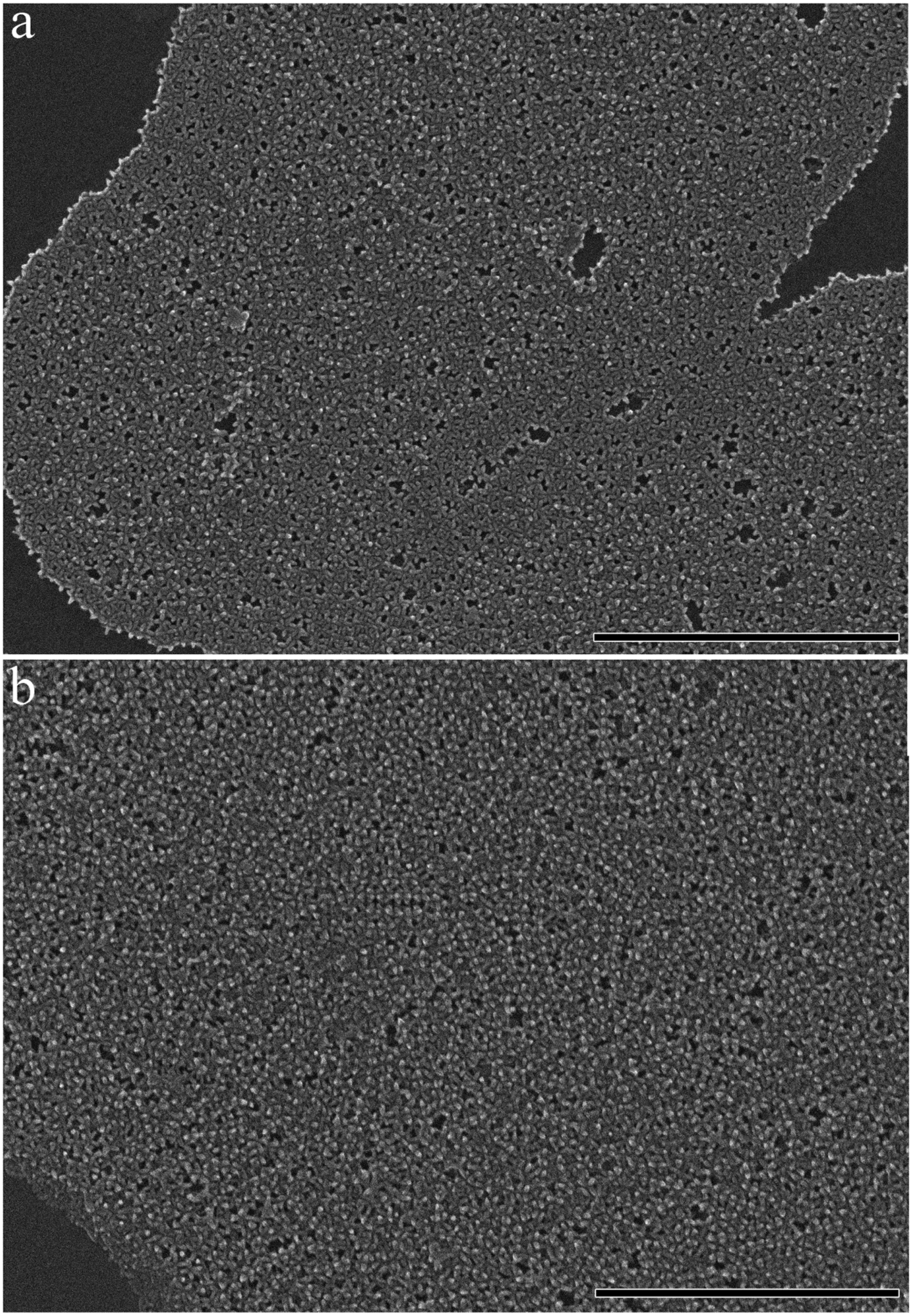

Supplementary Figure 9. Low-mag SEM view of the self-assembled planar superlattices formed by octapods with L/D of 5.0 (a) and 6.0 (b) extended over large regions. Scale bars: 1 μm.

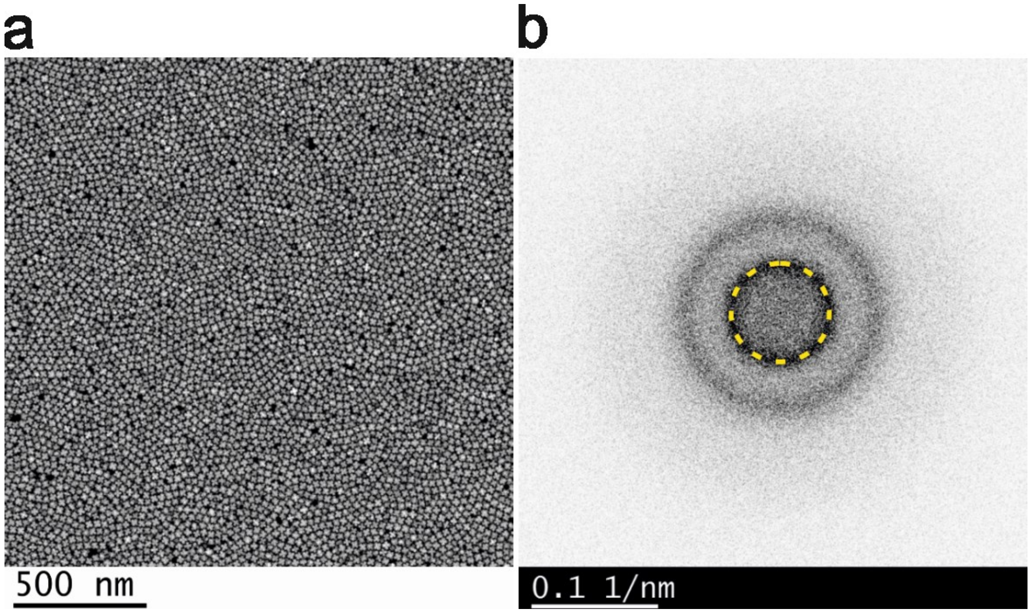

Supplementary Figure 10. Analysis of octapod-octapod spacing within the planar superlattices formed by octapods with an L/D of 3.0. (a) HAADF-STEM overview image of the superlattice. (b) Inverted FFT calculated from the same image, clearly showing a constant nearest-neighbour distance (with an calculated value of ca. 25.4 nm) between the particles (dashed yellow circle).

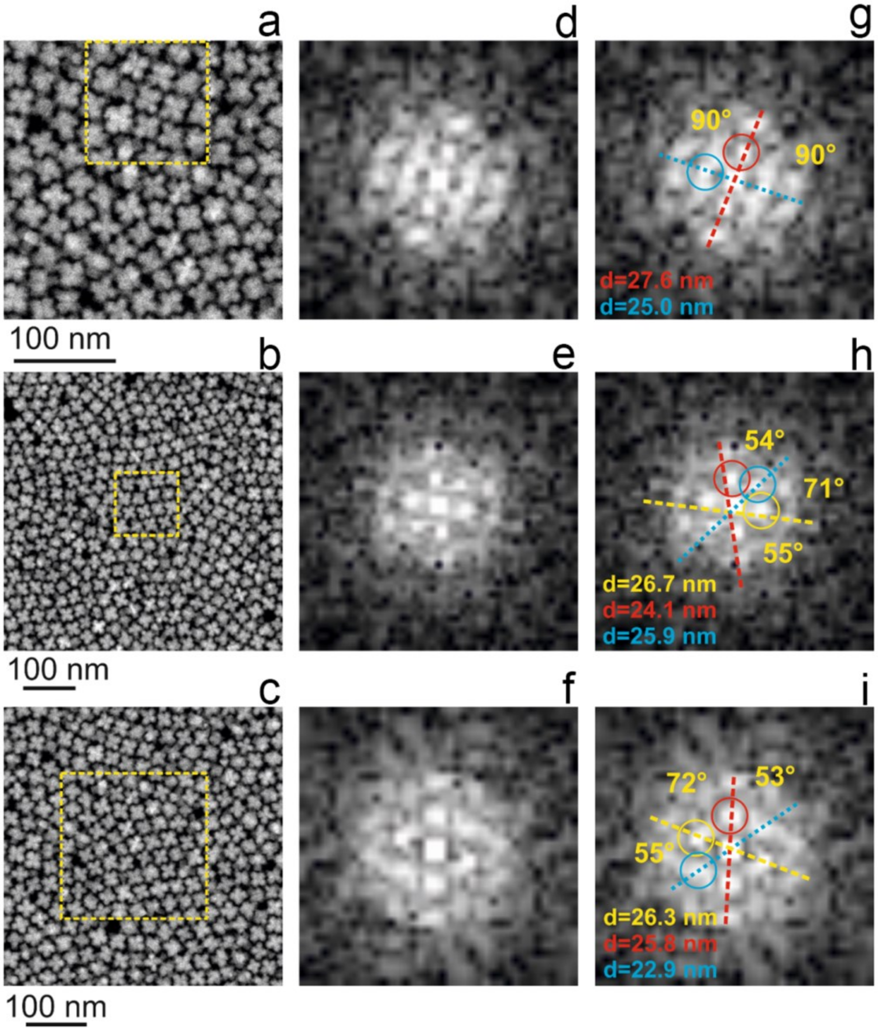

Supplementary Figure 11. Analysis of the configuration of octapods in three different small domains of the planar superlattices formed by octapods with an L/D of 3.0: (a-c) HAADF-STEM images showing the selected domains (framed in yellow). (d-f) Corresponding FFT of the selected domain. In (g-i) the respective FFT with indication of distances corresponding to spots and angles. The square geometry is observed in small domains (g), while different geometries are found in other regions due to the different alignments of pods between closer octapods (h, i).

Supplementary Note 4: Experimentally varying the octapod density

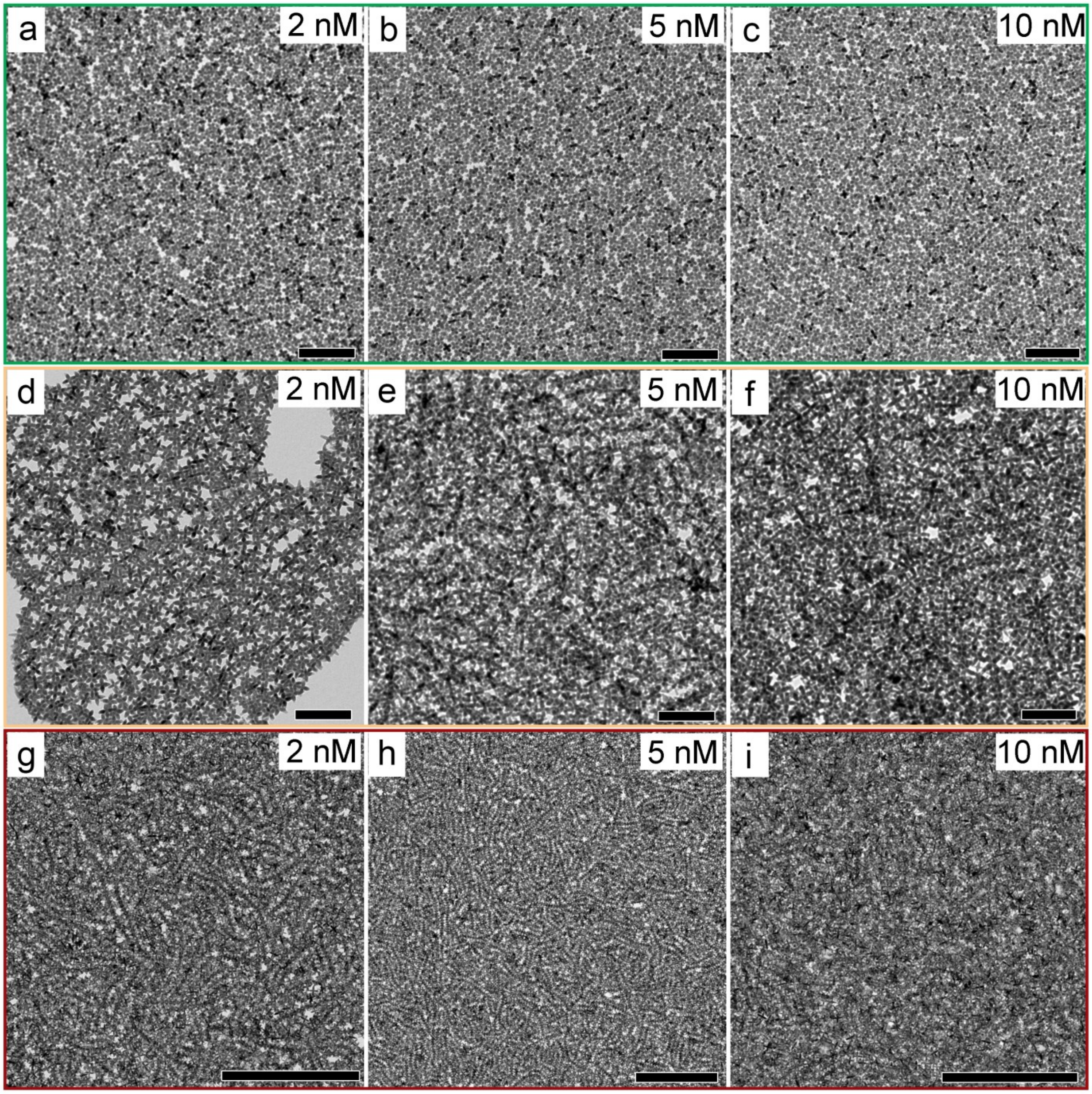

Supplementary Figure 12. Effect of variations on the density of octapods, from 2nM to 10 nM, on their configuration in 2D superlattices: (a-c) for L/D = 3.5 (green framed panels); (d-f) L/D = 5.0 (orange framed panels); and (g-i) L/D = 6.0 (red framed panels). The specific density of octapods used in the assembly is provided in the inset on each panel. Scale bars for the first two rows: 200 nm; scales bars for the last row: 500 nm, respectively.

At low octapod density (2 nM), the interlocked configuration is suppressed in octapods with $L/D = 5.0$ (Supplementary Fig. 12d), while it is retained at higher concentrations (10 nM), albeit in shorter segments (Supplementary Fig. 12f). This is attributed to an oversaturated condition reached at 10 nM that limits octapod out-plane mobility. In the case of octapods with

L/D = 6.0, the ability to form interlocked structures remains even at the lowest concentration used in the experiments (Supplementary Fig. 12g-i). For this concentration, we observe domains formed by interconnected chains. These structures exhibit a different projection from the classical interlocked one (Supplementary Fig. 13)

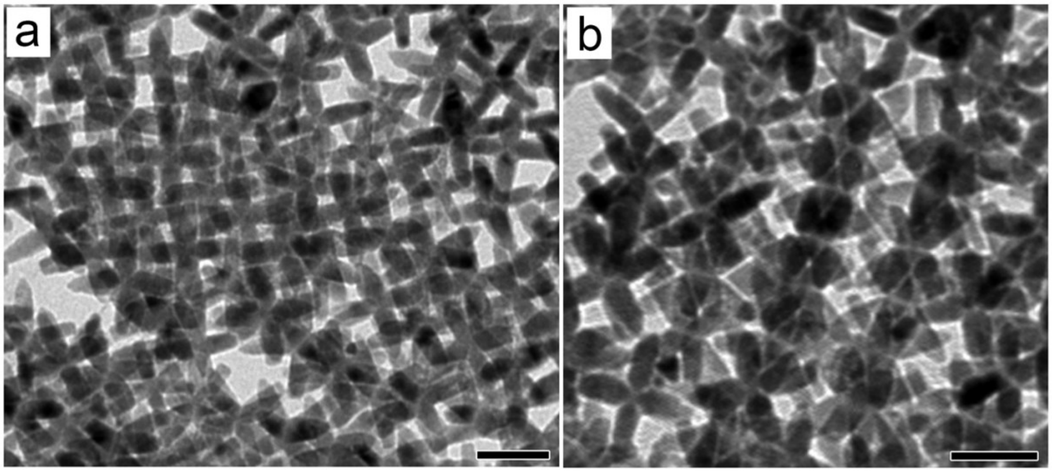

Supplementary Figure 13. Close-up view of the packing of chains observed when changing octapod density in particles with L/D of 6.0: Domains composed of well-interconnected chains observed at a low particle concentration of 2 nM forming a sort of 2D fabric (a) and 2D packing of classic interlocked chains of octapods observed at a particle concentration of 5 nM (b). Scale bars: 50 nm.

Supplementary Note 5: Surface analysis of as-synthesized octapods via FTIR.

Ligand type	Molecule	Number P/CH_3	$(CH_2/CH_3)_{FG}$ ratio
Hexylphosphonic acid, HPA	$CH_3(CH_2)_4CH_2-P(=O)(OH)-OH$	1	5
Octadecylphosphonic acid, ODPA	$CH_3(CH_2)_{16}CH_2-P(=O)(OH)-OH$	1	17
Trioctylphosphine, TOP	$CH_3(CH_2)_6CH_2-P(CH_2(CH_2)_6CH_3)_2$	0.3	7
Trioctylphosphine oxide, TOPO	$CH_3(CH_2)_6CH_2-P(=O)(CH_2(CH_2)_6CH_3)-CH_2(CH_2)_6CH_3$	0.3	7

Supplementary Table 2. Phosphorus-based ligands used in all the syntheses of octapods. The $(CH_2/CH_3)_{FG}$ ratio reported in the last column corresponds to the number of methylene functional group present in the pure ligand molecule per each methyl group.

It should be noted that, given the crystal structure of octapods[4], the shafts expose both Cd and S ions. Thus, TOP (bound to S ions) and the phosphonic acids (HPA and ODPA, that form complexes with Cd^{2+} complex in the synthesis) can be both localized on the shafts. For the tip surfaces, instead, the presence of TOP would exclude the presence of acids, since the tips can be either Cd or S-terminated[4, 7].

From the FTIR spectra shown in Fig. 3a of the main text, we identify four main peaks in the region from 3000 cm^{-1} up to 2800 cm^{-1} that contains the CH_3 and CH_2 symmetric and asymmetric stretching modes from the alkyl tails of the surface bounded ligand molecules[8]; and four main peaks in the region from 1200 cm^{-1} up to 900 cm^{-1} (named as P_1, P_2, P_3, and P_4 in the spectra, $P_{n=1\ldots4}$) that are assigned to the P-O(H) and P=O stretching modes derived from the ligand's polar heads[9-12]. In detail:

3000-2800 cm^{-1}: collection of the CH_2 and CH_3 stretching peaks deriving from the alkyl chain(s) present in all the phosphorus-based bonded ligands. Namely:

2957 cm^{-1} CH_3 asymmetric stretching;
2923 cm^{-1} CH_2 asymmetric stretching;
2872 cm^{-1} CH_3 symmetric stretching (shoulder);
2854 cm^{-1} CH_2 symmetric stretching;

1250-850 cm^{-1}: peaks related to the P-O stretching modes deriving from the polar head of the ligands formed by a P=O moiety (TOPO or phosphonic acids) or by a PO_3^{2-} (HPA and ODPA). The peaks can be identified in this way following[13]:
P_1 at 1167 cm^{-1} P=O stretching from acids;
P_2 at 1100 cm^{-1} P=O stretching from TOPO and HPA;
P_3 at 1054 cm^{-1} (broad shoulder) P=O from acids;
P_4 at 926 cm^{-1} P-OH stretching from acids.

On the other hand, to elucidate the role of the phosphonic acids in the synthesis of octapods, we conducted experiments in which only one ligand was included in the protocol, HPA or ODPA. As can be appreciated in Supplementary Fig. 11, the presence of only ODPA in the

synthesis along with TOP and TOPO leads to tetrapods and short CdS rods (Supplementary Fig. 14a). The mixture of HPA+TOP+TOPO promotes the growth of structures with more than four pods (Supplementary Fig. 14b). Note that ODPA appears to stabilize flat-terminated pods (see blue-framed tetrapod in a), while sharp tips are observed in the multipod's structures obtained when only HPA+TOP+TOPO is used in the synthesis (see cyan-framed structures in b).

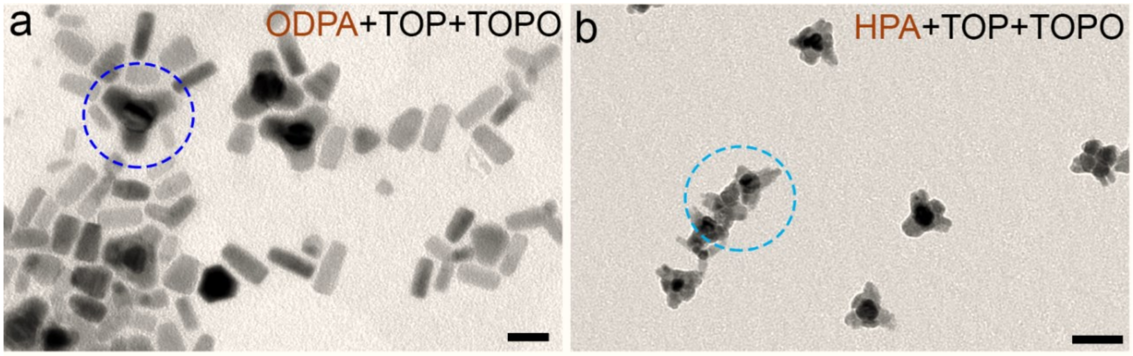

Supplementary Figure 14. TEM images of the resulting nanocrystals obtained when a single phosphonic acid is used in the synthesis of octapods with L/D of 6.0: (a) only ODPA and (b) only HPA. Scales bars: 20 nm (a) and 50 nm (b), respectively.

To identify further the role of the short ligands (HPA and TOP), control experiments were carried out, wherein we reduced the amount of TOP employed in the synthesis of octapods with L/D of 5.0, see Supplementary Fig. 15. Eight pods can be observed in the formed structures, however, the octapod shape itself is compromised, see Supplementary Fig. 15b. Also, the resulting nanocrystals are smaller – estimated L/D of approx. 2.5 – compared to those synthesized with a higher amount of TOP (Supplementary Fig. 15a). The resulting structures have larger pod diameters (approx. 20 nm) and shorter pod length (approx. 18 nm), which suggest that TOP plays a key role in stabilizing the pod shafts, especially at the beginning of their growth.

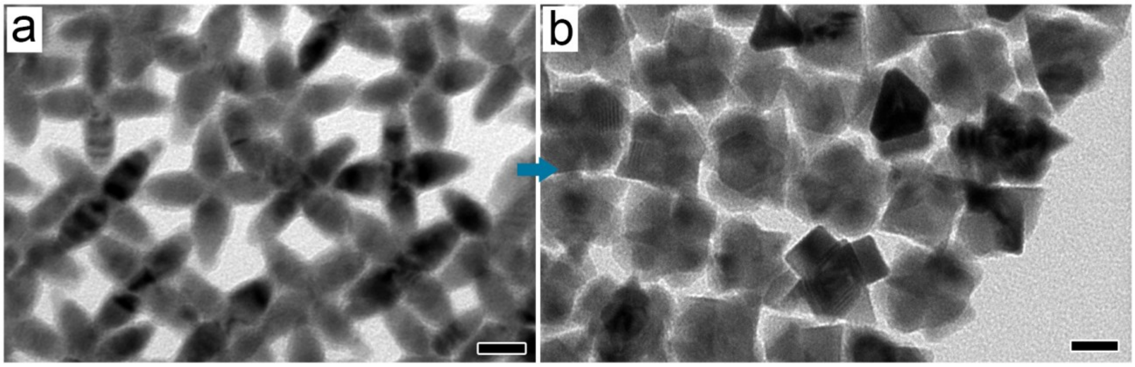

Supplementary Figure 15. (a) TEM image of octapods with an L/D of 5.0 (prepared with 0.4 nmol of $Cu_{2-x}Se$ NCs) and (b) particles obtained using a reduced amount of TOP (only 0.5 ml of TOP and 0.62 ml of TOPS injected at 350°C) in the synthesis of the same octapods. Scale bars: 20 nm.

Supplementary Note 6: The model used in the Monte Carlo simulations of the octapod self-assembly

In this note, we provide additional detail of our simulation model. We use four intersecting spherocylinders with a hard overlap potential to represent the excluded volume interactions between the octapods. The shaft – the part that does not include the hemispherical caps – has length L' ($\mathrm{L}'/2 = P_l$) and the diameter is given by D, so that $\mathrm{L} = \mathrm{L}' + \mathrm{D}$ and $\mathrm{L}/\mathrm{D} = \mathrm{L}'/\mathrm{D} + 1$, see Supplementary Fig. 16.

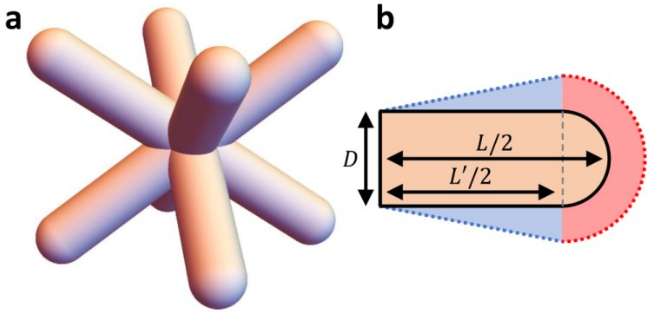

Supplementary Figure 16. The simulation model that reproduces the self-assembled structures found in the experiment. (a) A three-dimensional (3D) representation of the hard-core of the octapod model, comprised of four intersecting spherocylinders; $L/D = 7$ in this case. (b) Visualization of the shaft length $L'/2$, the shaft diameter D, and the length of the pod, $L/2$. The soft interactions that the pods are endowed with are sketched as well. The red-colored region indicates the range of the tip potential. The blue-colored region indicates that the potential is weaker towards the core of the octapod.

We endow this model with short-ranged (soft) interaction potentials to account for the interaction between the ligands coating the octapods. Here, we distinguish between the way the shaft and tip of each pod interacts with one another, since the experiments suggest a difference in ligand coating between these parts of the pods. A sketch of the interactions is given in Supplementary Fig. 16b. Centered on each tip, we have an isotropic square-well attraction which interacts only with other tips

$$U_{\mathrm{SW,tip}}(r) = \begin{cases} \varepsilon_{\mathrm{T}}, & r < r_c \\ 0, & r \geq r_c \end{cases},$$

where r is the distance between two tips, r_c is the cut-off distance, and ε_{T} is the strength of attraction ($\varepsilon_{\mathrm{T}} < 0$) or repulsion ($\varepsilon_T > 0$) between the tips in terms of $k_{\mathrm{B}}T$, with k_{B} Boltzmann's constant and T the temperature. We chose $r_c = (3/2)D$, since the diameter of the octapods is approximately 10 nm (for the larger values of L/D), and twice the ligand length is approximately 5 nm, meaning that when the distance between two pods exceeds 15 nm there is no interaction.

The shafts additionally interact via an anisotropic square well potential, which is given by

$$U_{\mathrm{SW,shaft}}(d,R,\hat{u},\hat{v}) = \begin{cases} \varepsilon_{\mathrm{S}} \frac{R}{L'}\left(1+\frac{L'}{D}\left(1-\frac{R}{L'}\right)|\hat{u}\cdot\hat{v}|\right), & d<r_c \cap R<L' \\ 0, & \text{otherwise} \end{cases},$$

where ε_{S} is the interaction strength between the shafts. Here, d is the minimum distance between the two shafts, as computed via the standard distance criterion for two spherocylinders,[14] which is also used to determine the excluded-volume interaction. The distance R is measured between the centers of the octapods, and $\hat{u}$ and $\hat{v}$ are unit vectors that describe the orientations of the respective shafts.

The expression for the shaft interaction may look complicated, but is relatively straightforward to understand. i) The condition $R < L'$ ensures that there is only a contribution from $U_{\mathrm{SW,tip}}$, when two octapods are spaced and oriented in such a way that only two of the tips come closer than r_c. ii) The weighting term R/L' is used to model the experimental observation that the ligand coating is sparser towards the core of the octapod. We hypothesized this to mean that the interaction effected by these ligands is weaker closer to the core, which is captured by the linear decay with core-core distance. This decay is weighted with the octapod shaft length to give 1, when the two octapods are spaced exactly L' apart. That is, the maximum value is achieved before the transition to tip interactions is made, in the situation that the two pods slide along and away from each other. iii) The second term in the brackets is used to account a stronger interaction when two shafts are aligned; we use the absolute value of the dot-product between the orientation vectors as a measure of this alignment. The factor $(1 - R/L')$ accounts for the amount of overlap when two pods are aligned, which vanishes when the octapods are spaced roughly L' apart. iv) The factor L'/D is a weight used to differentiate between the strength of aligned interaction, which is proportional to the contact area $L'D$, and the strength of misaligned interaction, which is proportional to D^2.

It should be noted that is a very simple model with effective interactions for a physically complicated system. Preliminary investigations that did not account for shaft alignment or did not differentiate between tips and shafts, did not produce configurations that were representative of the experimental findings.

Supplementary Note 7: Additional supplementary figures from the Monte Carlo simulations

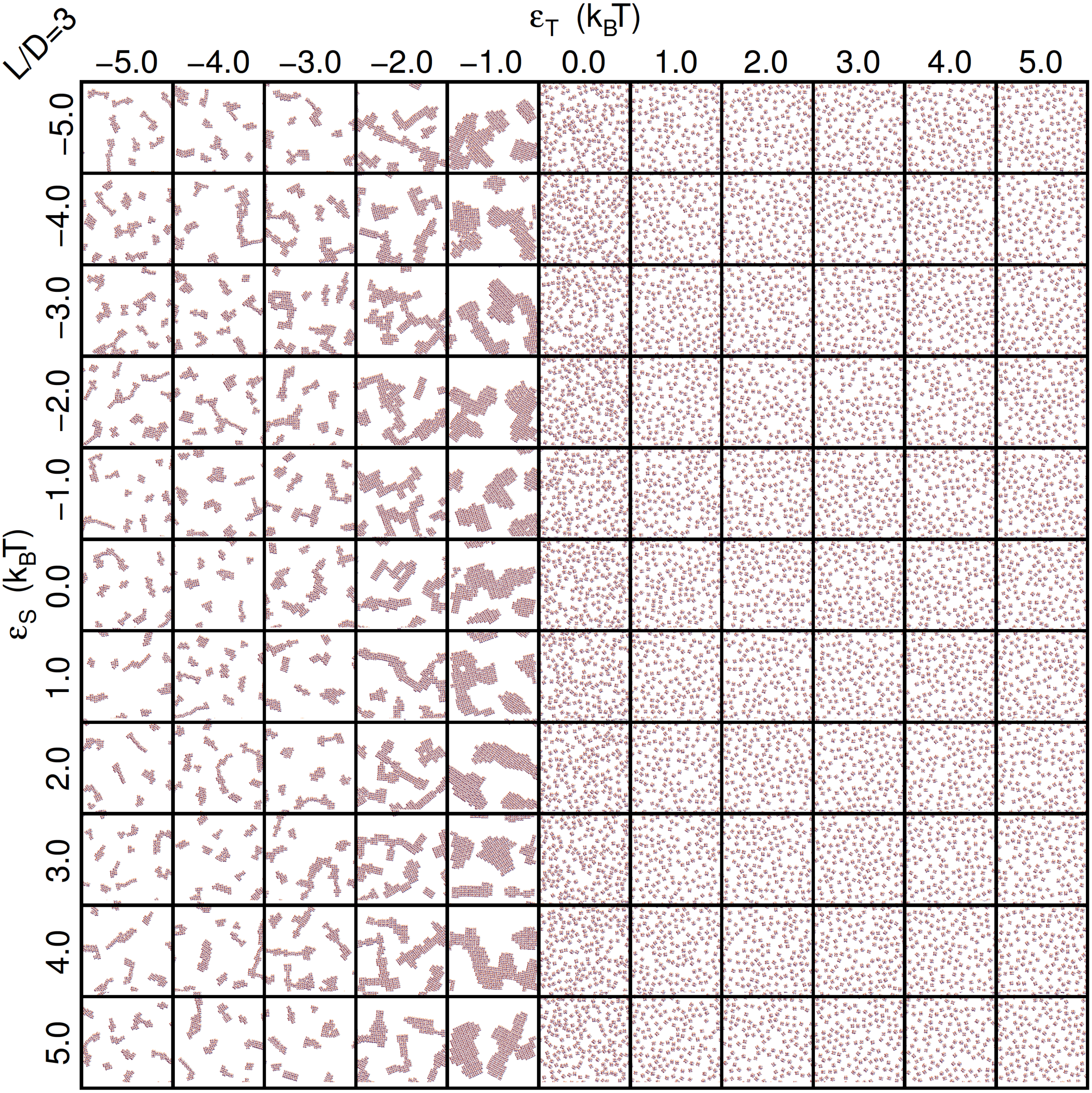

Supplementary Figure 17. Snapshots showing a typical top-view configuration of octapods at the end of our simulation run, for an aspect ratio L/D of 3.0. The snapshots are shown as a function of the shaft interaction parameter ε_S (from top to bottom) and the tip interaction parameter ε_T (from left to right), both expressed in terms of $k_B T$.

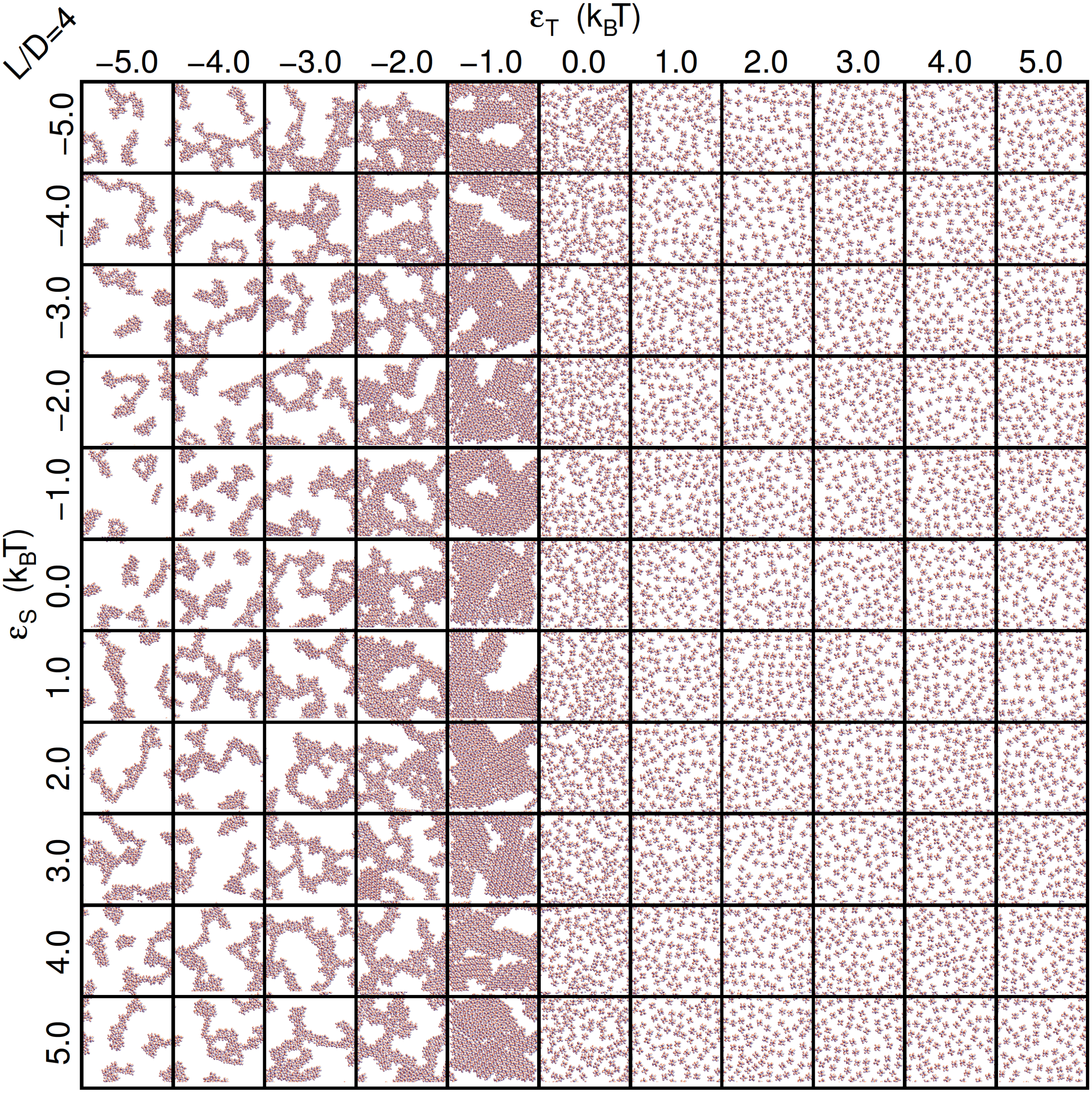

Supplementary Figure 18. Snapshots showing a typical top-view configuration of octapods at the end of our simulation run, for an aspect ratio L/D of 4.0. The snapshots are shown as a function of the shaft interaction parameter ε_S (from top to bottom) and the tip interaction parameter ε_T (from left to right), both expressed in terms of k_BT.

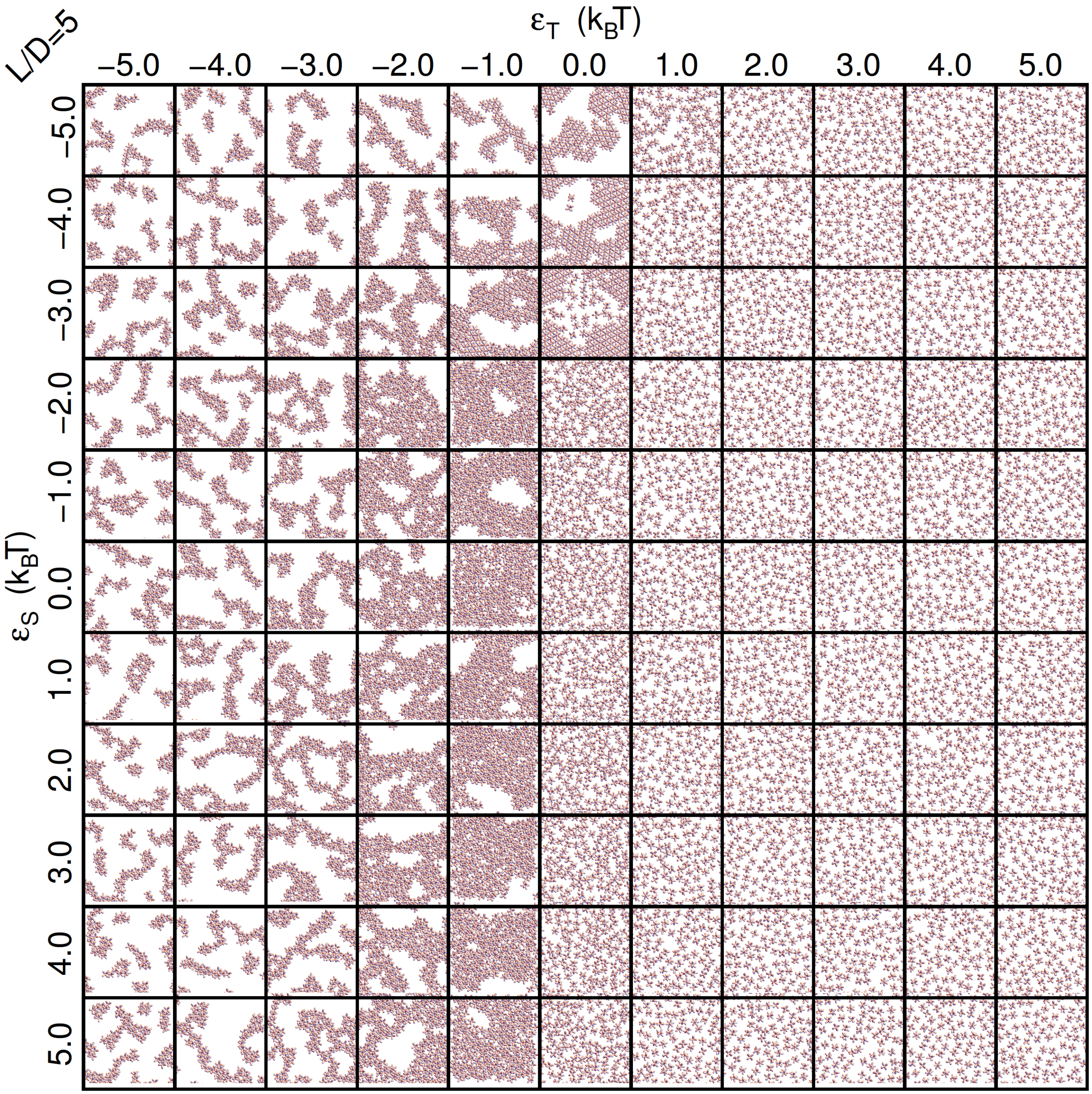

Supplementary Figure 19. Snapshots showing a typical top-view configuration of octapods at the end of our simulation run, for an aspect ratio L/D of 5.0. The snapshots are shown as a function of the shaft interaction parameter ε_S (from top to bottom) and the tip interaction parameter ε_T (from left to right), both expressed in terms of k_BT.

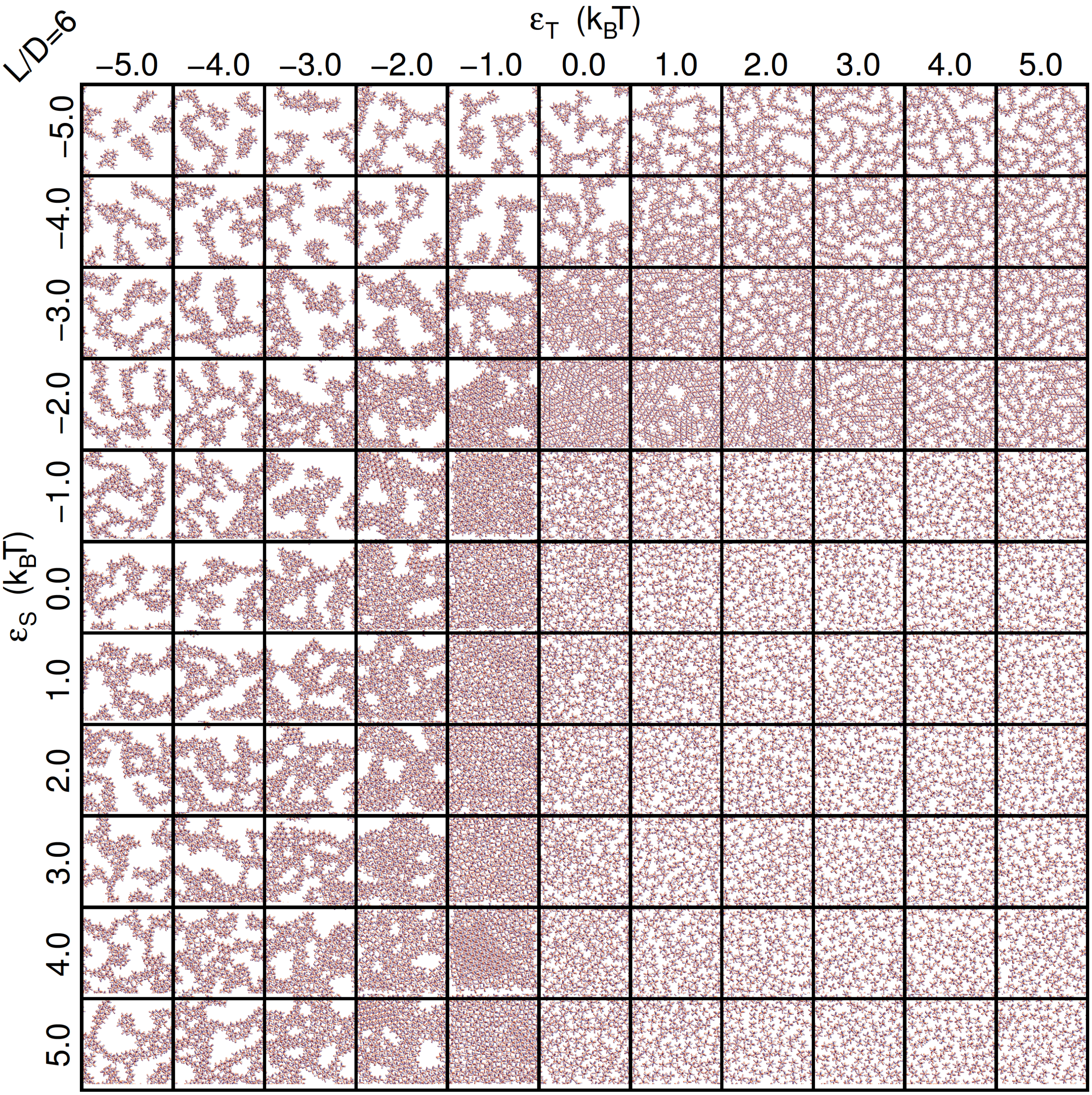

Supplementary Figure 20. Snapshots showing a typical top-view configuration of octapods at the end of our simulation run, for an aspect ratio L/D of 6.0. The snapshots are shown as a function of the shaft interaction parameter ε_S (from top to bottom) and the tip interaction parameter ε_T (from left to right), both expressed in terms of $k_B T$.

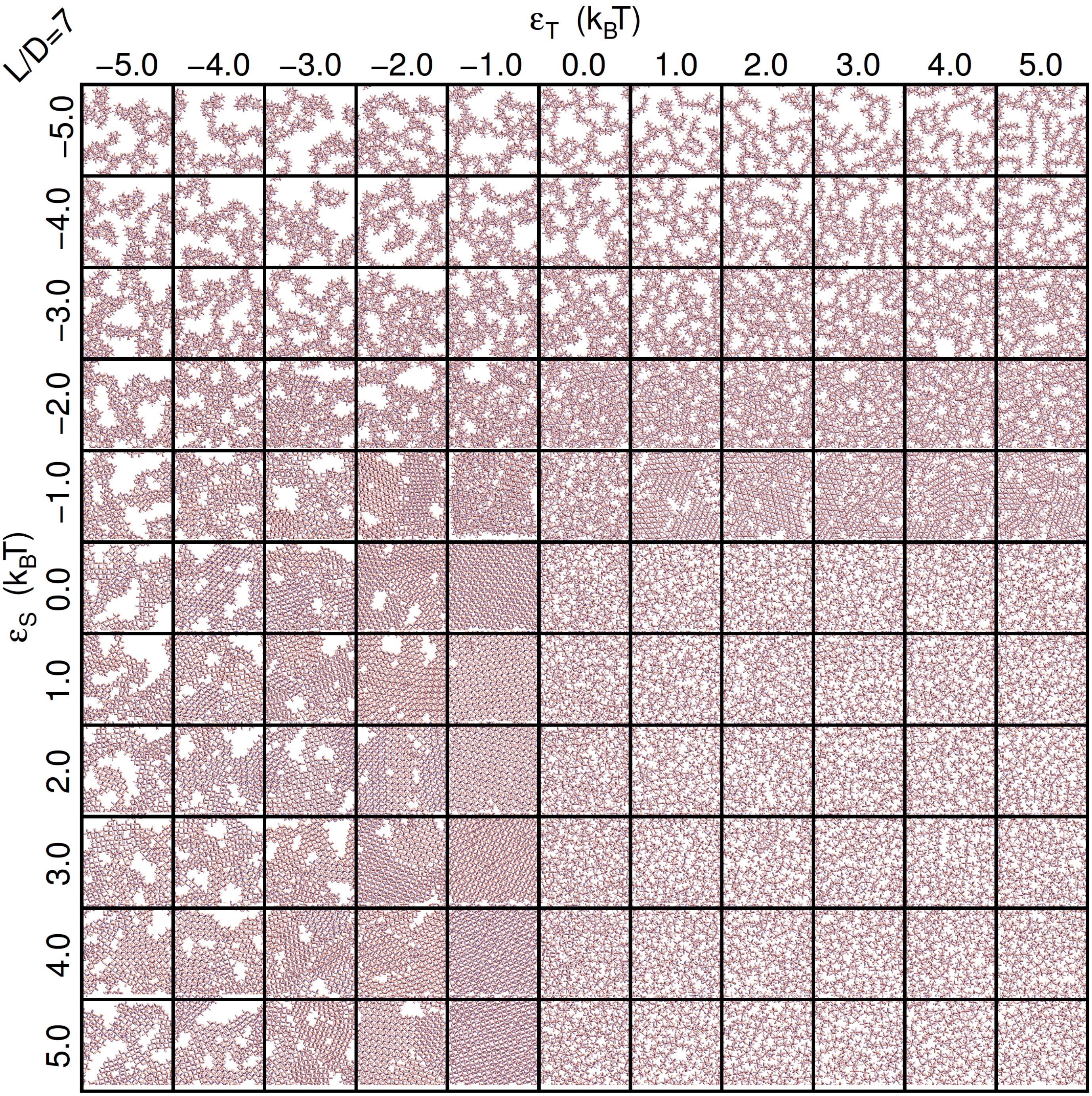

Supplementary Figure 21. Snapshots showing a typical top-view configuration of octapods at the end of our simulation run, for an aspect ratio L/D of 7.0. The snapshots are shown as a function of the shaft interaction parameter ε_{S} (from top to bottom) and the tip interaction parameter ε_{T} (from left to right), both expressed in terms of $\mathrm{k_B T}$.

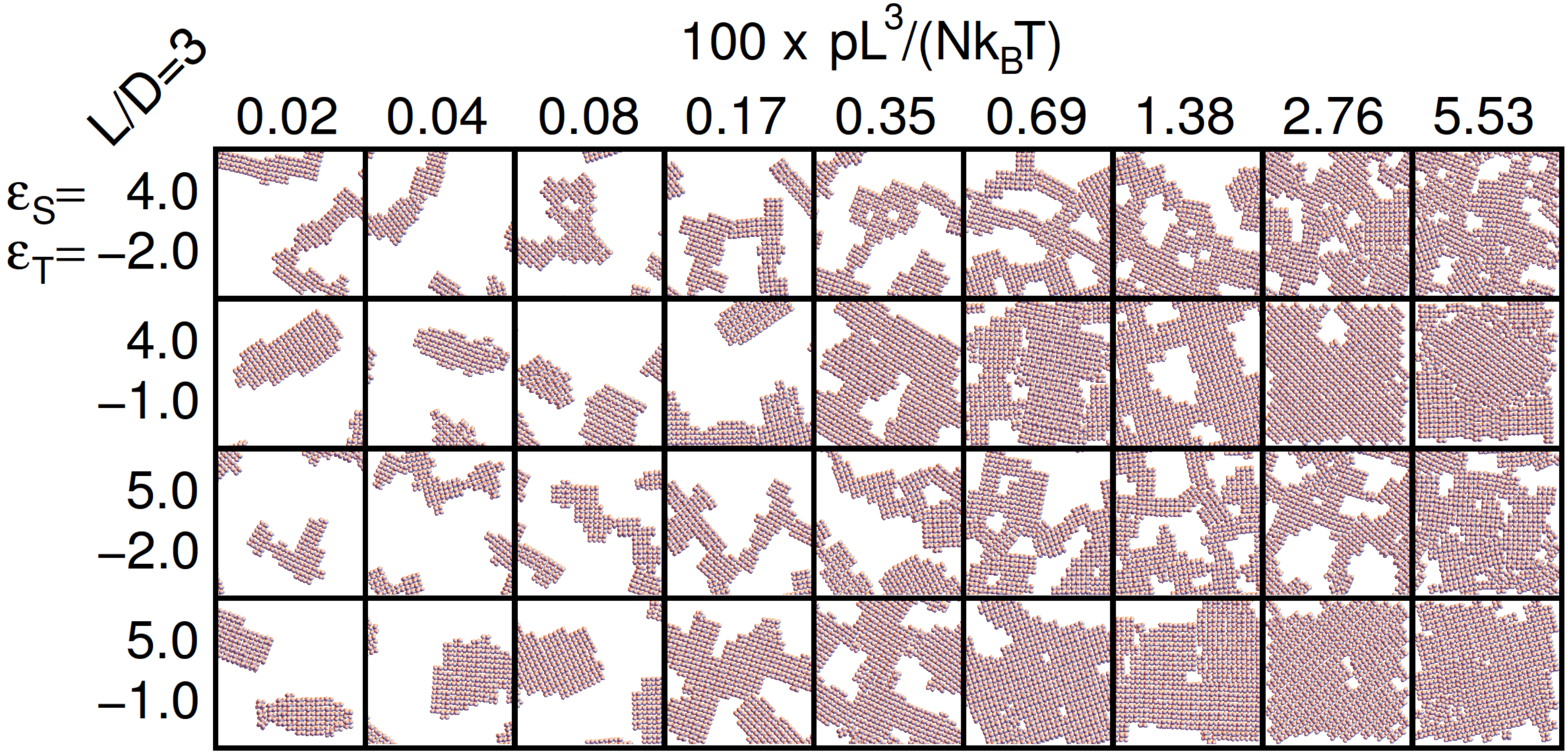

Supplementary Figure 22. Snapshots showing a typical top-view configuration of octapods with an aspect ratio L/D of 3.0 at the end of our simulation run for selected values of the shaft interaction parameter ε_S and the tip interaction parameter ε_T, both expressed in terms of $k_B T$, as provided on the left-hand side. The aspect ratio of L/D is again given in the top-left corner. The pressure p is increased from left to right and made dimensionless by Nk_BTL^{-3}. The pressure values have been multiplied with a factor of 100, so a value of 1.0 corresponds to $0.01Nk_BTL^{-3}$.

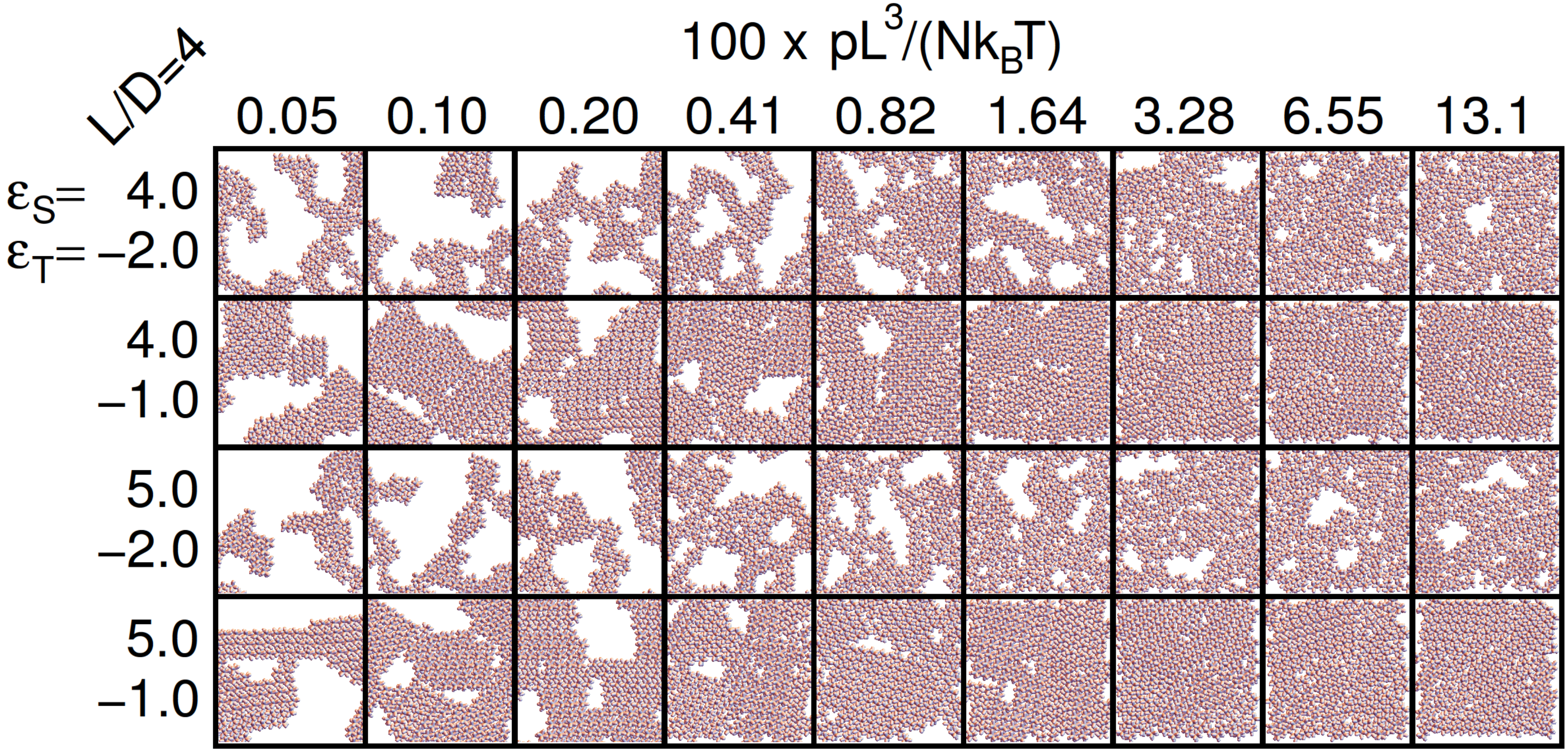

Supplementary Figure 23. Snapshots showing a typical top-view configuration of octapods with an aspect ratio L/D of 4.0 at the end of our simulation run for selected values of the shaft interaction parameter ε_S and the tip interaction parameter ε_T, both expressed in terms of $k_B T$, as provided on the left-hand side. The aspect ratio of L/D is again given in the top-left corner. The pressure p is increased from left to right and made dimensionless by $Nk_B TL^{-3}$. The pressure values have been multiplied with a factor of 100, so a value of 1.0 corresponds to $0.01 Nk_B TL^{-3}$.

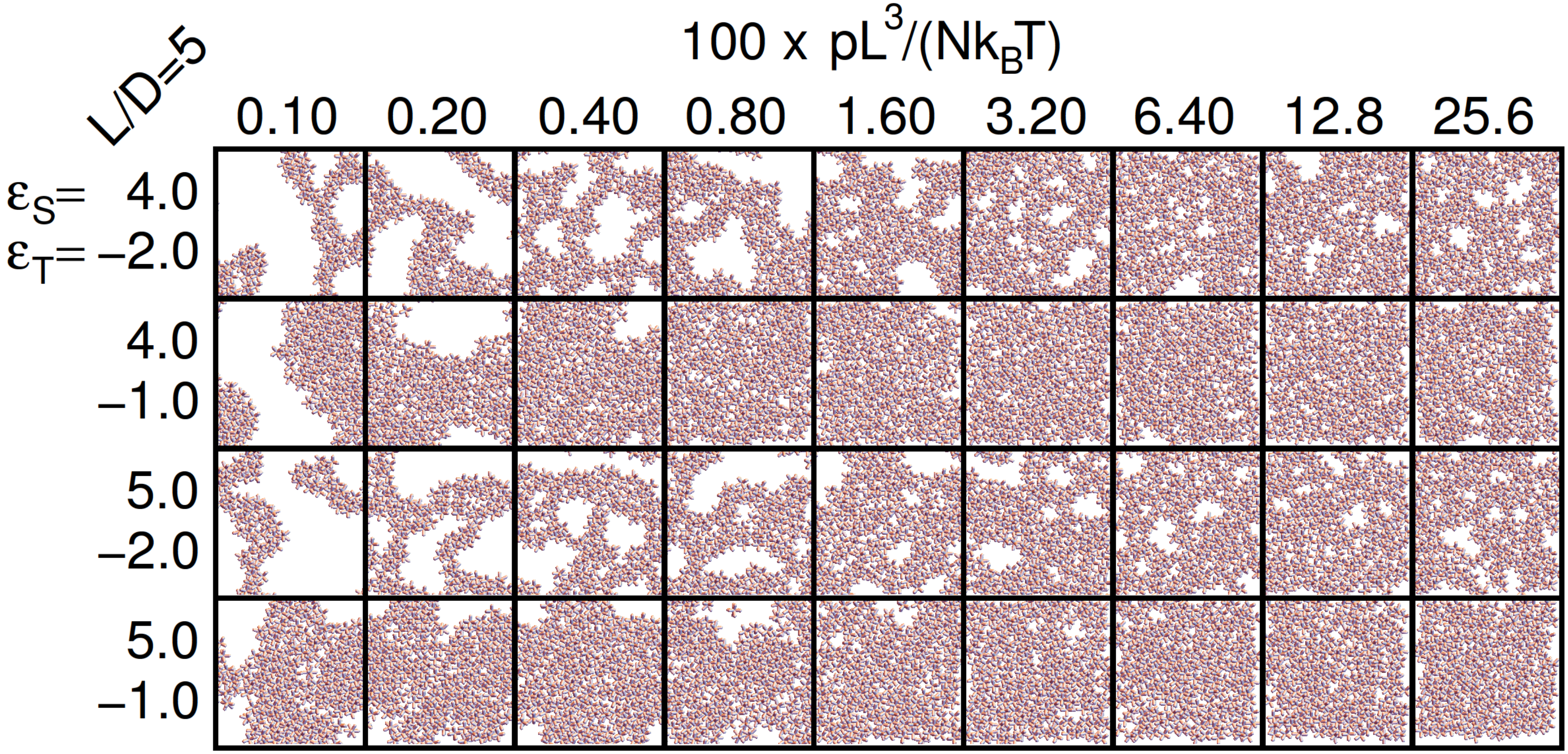

Supplementary Figure 24. Snapshots showing a typical top-view configuration of octapods with an aspect ratio L/D of 5.0 at the end of our simulation run for selected values of the shaft interaction parameter ε_S and the tip interaction parameter ε_T, both expressed in terms of k_BT, as provided on the left-hand side. The aspect ratio of L/D is again given in the top-left corner. The pressure p is increased from left to right and made dimensionless by Nk_BTL^{-3}. The pressure values have been multiplied with a factor of 100, so a value of 1.0 corresponds to $0.01Nk_BTL^{-3}$.

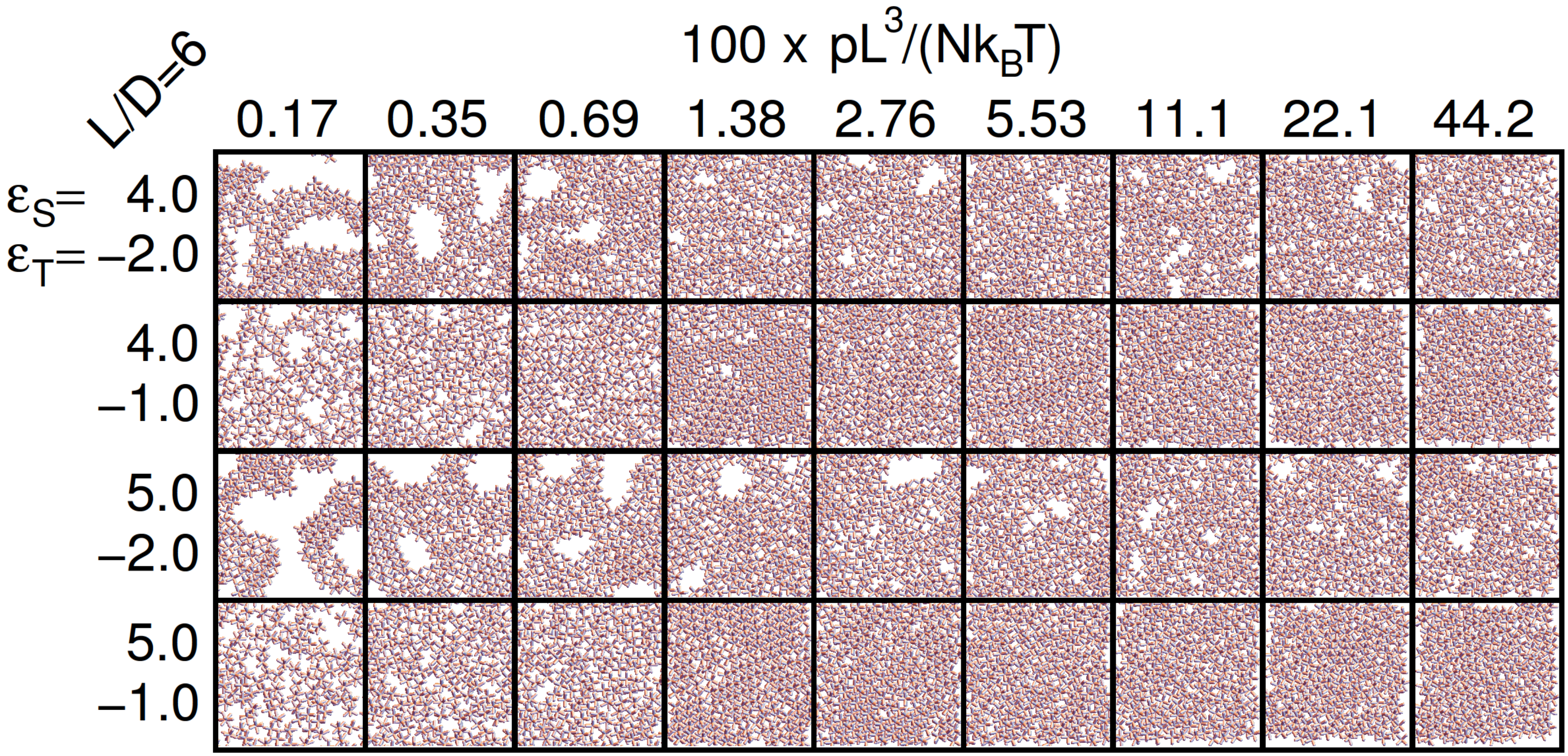

Supplementary Figure 25. Snapshots showing a typical top-view configuration of octapods with an aspect ratio L/D of 6.0 at the end of our simulation run for selected values of the shaft interaction parameter ε_S and the tip interaction parameter ε_T, both expressed in terms of $k_B T$, as provided on the left-hand side. The aspect ratio of L/D is again given in the top-left corner. The pressure p is increased from left to right and made dimensionless by $Nk_B TL^{-3}$. The pressure values have been multiplied with a factor of 100, so a value of 1.0 corresponds to $0.01Nk_B TL^{-3}$.

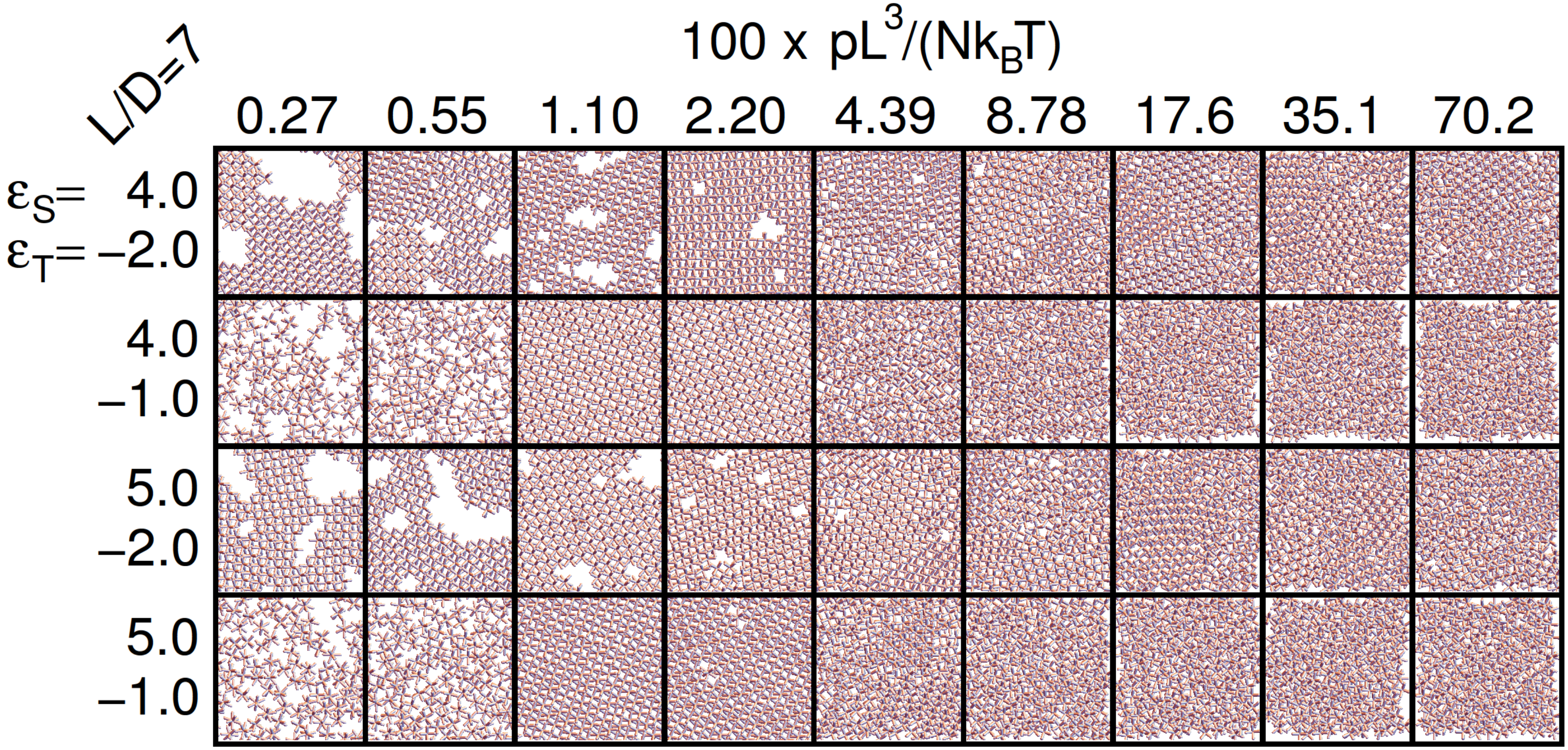

Supplementary Figure 26. Snapshots showing a typical top-view configuration of octapods with an aspect ratio L/D of 7.0 at the end of our simulation run for selected values of the shaft interaction parameter ε_S and the tip interaction parameter ε_T, both expressed in terms of $k_B T$, as provided on the left-hand side. The aspect ratio of L/D is again given in the top-left corner. The pressure p is increased from left to right and made dimensionless by $Nk_B TL^{-3}$. The pressure values have been multiplied with a factor of 100, so a value of 1.0 corresponds to $0.01Nk_B TL^{-3}$.

Supplementary Note 8: Self-assembled planar superlattices from ligand-exchanged octapods.

Ligand type	Molecule	Number S/CH_3	$(CH_2\,/CH_3)_{FG}$ ratio
1-hexanethiol, HT	$CH_3(CH_2)_4CH_2SH$	1	5
1-dodecanethiol, DDT	$CH_3(CH_2)_{10}CH_2SH$	1	11
1-octadecanethiol, ODT	$CH_3(CH_2)_{16}CH_2SH$	1	17
Oleylamine, OLAM	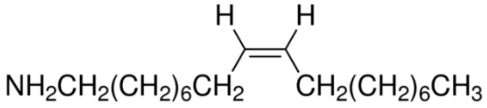	-	15

Supplementary Table 3. Molecules used in the ligand exchange of the as-synthesized octapods. The $(CH_2\,/CH_3)_{FG}$ ratio reported in the last column corresponds to the number of methylene functional group present in the pure ligand molecule per each methyl group.

The spectra collected from HT-, DDT-, and OLAM-ligand exchanged octapods (Supplementary Fig. 27) show a strong reduction of the $P_{n=1\ldots4}$ peaks (region between 1250 cm^{-1} and 850 cm^{-1}), related to the stretching modes of TOPO and the phosphonic acids (Supplementary Fig. 27). In contrast, the surfaces of the ODT-ligand exchanged octapods exhibit the same features observed for the as-synthesized octapods. A stronger CH_3 asymmetric stretching mode is presented by HT-exchanged particles, which indicates a thinner ligand shell compared to that of the DDT-exchanged octapods. We arrive at this conclusion by comparing the deconvoluted CH_2 and CH_3 asymmetric peaks (CH_2/CH_3 ratio) in the spectra collected from the HT- and DDT-exchanged octapods (Supplementary Fig. 28). For the OLAM-exchanged octapods (identified by the =C-H stretching mode signal at 2924 cm^{-1} and 2853 cm^{-1}), there is a reduction on the CH_3 asymmetric peak that is associated to a thicker ligand shell (Supplementary Fig. 28b); this was expected given OLAM's long chain (Supplementary Table 3). In the case of the TOP-exchanged octapods, their surfaces appear to be less rich of phosphonic acids, since only the signal coming from the P_1 peak remains in the spectrum, as for the as-synthesized particles. The surfaces of the TOPO-exchanged octapods, on the other hand, are still stabilized by phosphonic acids and are less rich of TOP, as evidenced by the increased signal of the P_n peaks compared to the CH_2 asymmetric stretching mode (Supplementary Fig. 27).

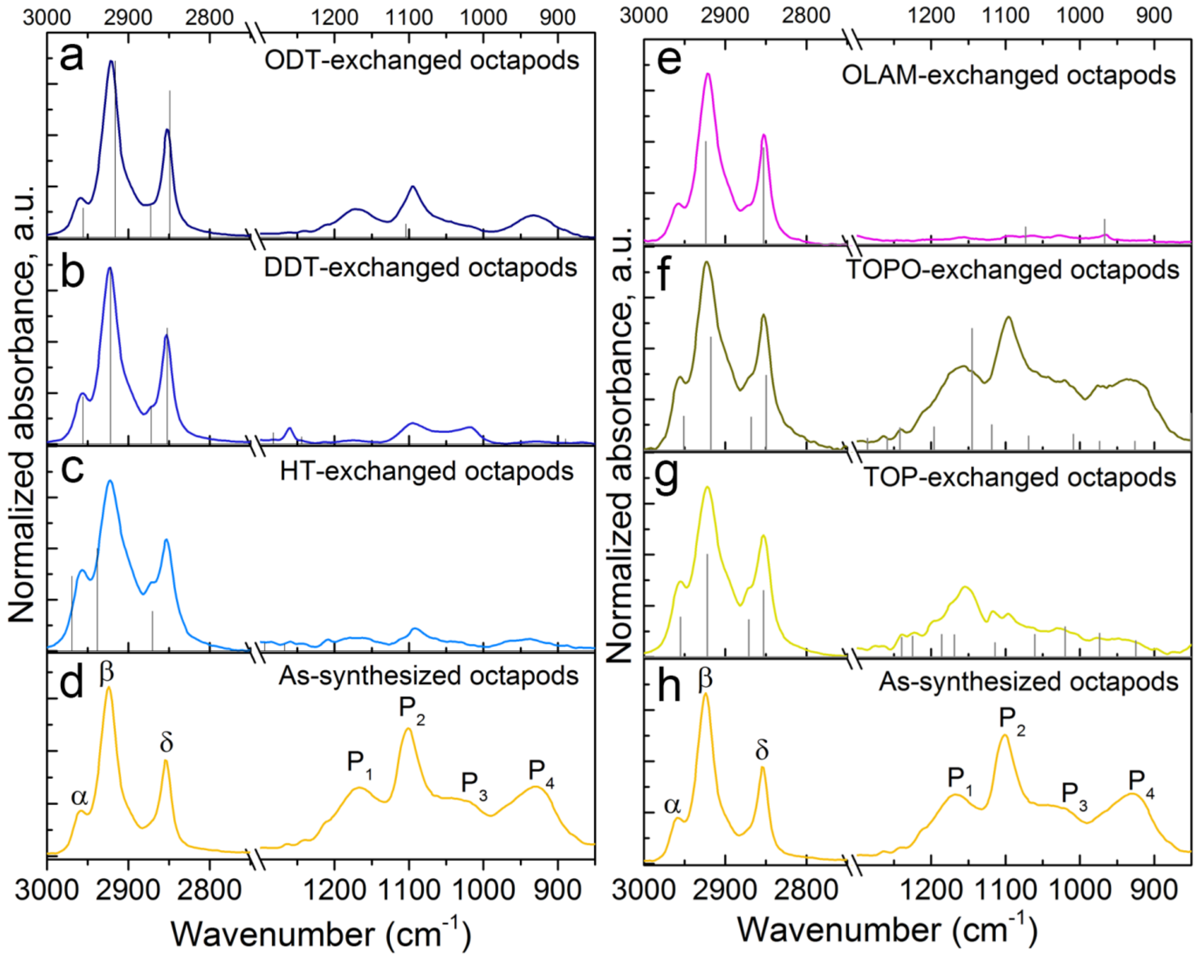

Supplementary Figure 27. FTIR spectra of octapods with L/D of 5.0 after ligand exchanges performed with different ligands: thiols (a-c), OLAM (e), TOPO (f), and TOP (g) evidencing the formation of new exchanged surfaces of octapods. The exception is the ODT ligand (a), for which no changes in the spectrum could be appreciated, when compared to that collected from as-synthesized octapods (d, h). The letters α, β, and δ embedded in the spectrum of the as-synthesized octapods represent the CH_3, CH_2 asymmetrical stretching modes and the CH_2 symmetrical stretching mode for the ligands, as described in the Supplementary Note 4. The FTIR spectra collected from the pure ligands are shown in grey vertical lines on each panel.

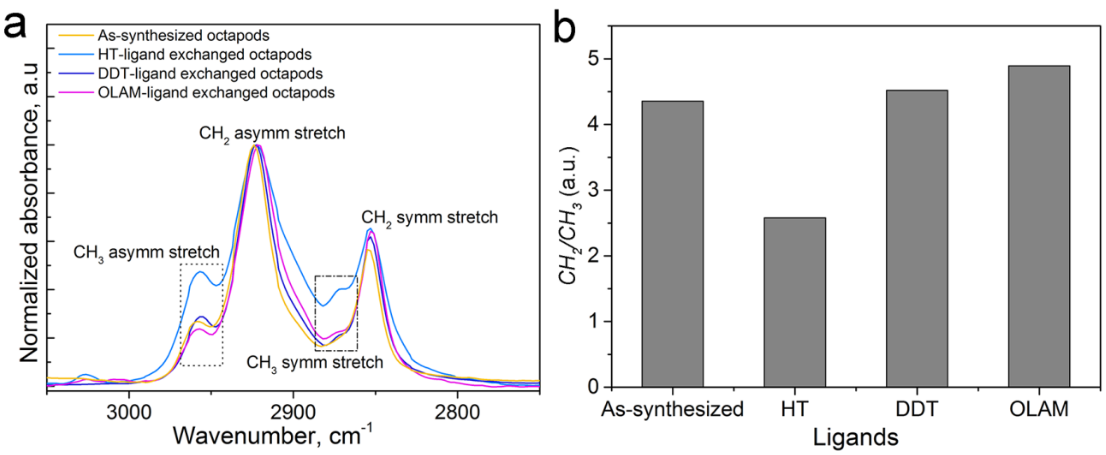

Supplementary Figure 28. (a) FTIR spectra of the as-synthesized and ligand exchanged octapods with L/D of 5.0 showing the region between 3000 cm^{-1} and 2700 cm^{-1}. (b) $\mathrm{CH_2/CH_3}$ratio between the deconvoluted CH_2 and CH_3 asymmetric stretching peaks calculated for the different exchanged octapods.

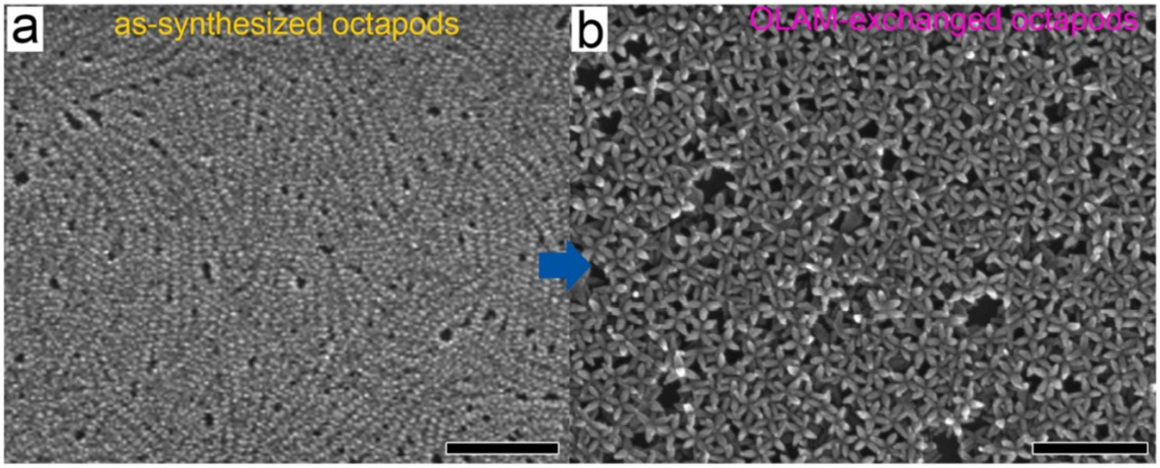

Supplementary Figure 29. SEM image of the planar superlattices formed by as-synthesized octapods with L/D of 6.0 exhibiting interlocked-chains (a), a configuration that is fully broken from the OLAM-exchanged particles (b). the Scale bars: 500 nm (a); 200 nm (b).

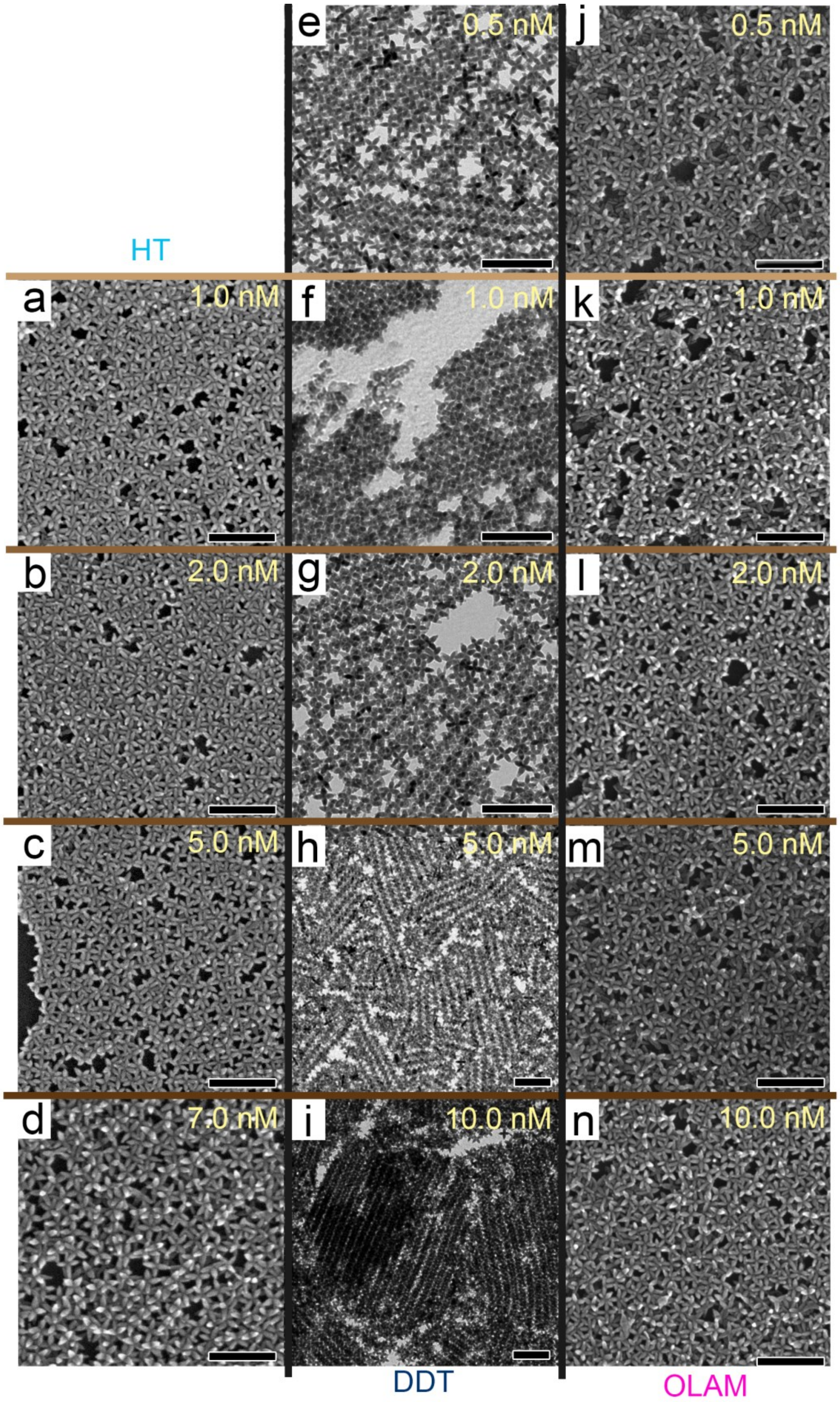

Supplementary Figure 30. Planar superlattices of (a-d) HT-, (e-i) DDT-, and (j-n) OLAM-exchanged octapods with L/D of 5.0 at different particle's densities, from 2 nM to 10 nM. Scale bars: 200 nm. In HT-exchanged particles, the concentration of octapods has a marked influence on their assembly, while, when the nanocrystals are coated by longer ligands (OLAM and DDT), their organization is not affected by concentration, as in the case of particles with lower L/D.

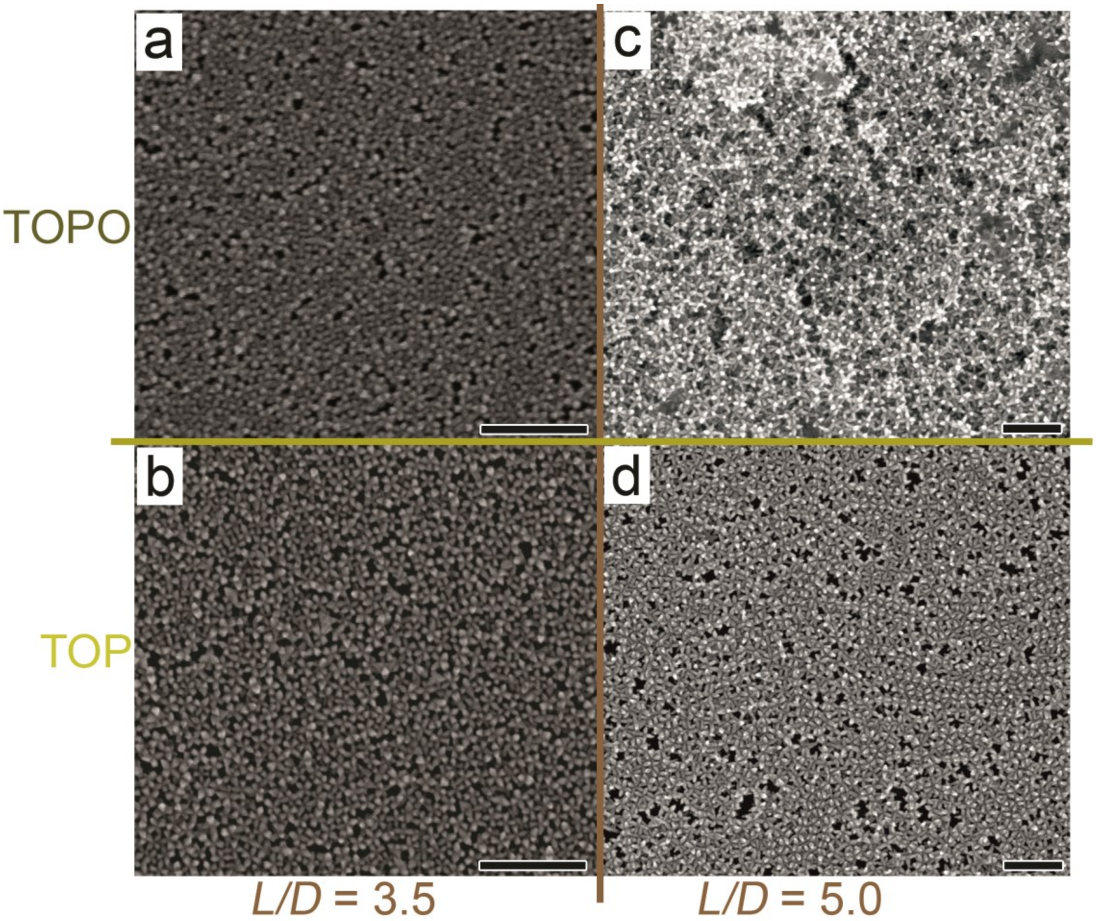

Supplementary Figure 31. SEM images of the planar superlattices formed by phosphine/phosphine oxide-exchanged octapods with L/D of 3.5 (a,b) and 5.0 (c,d) at 5 nM of octapods. Scale bars: 500 nm. As in the case of thiol-exchanged particles, octapods with a low L/D do not show variations on their assembly (a,b). TOPO-exchanged particles with a high L/D, instead, exhibit a strong aggregation (c). TOP-exchanged octapods with longer pods preserve their interlocked-chain packing, which is accompanied by regions of near-square lattices (d).

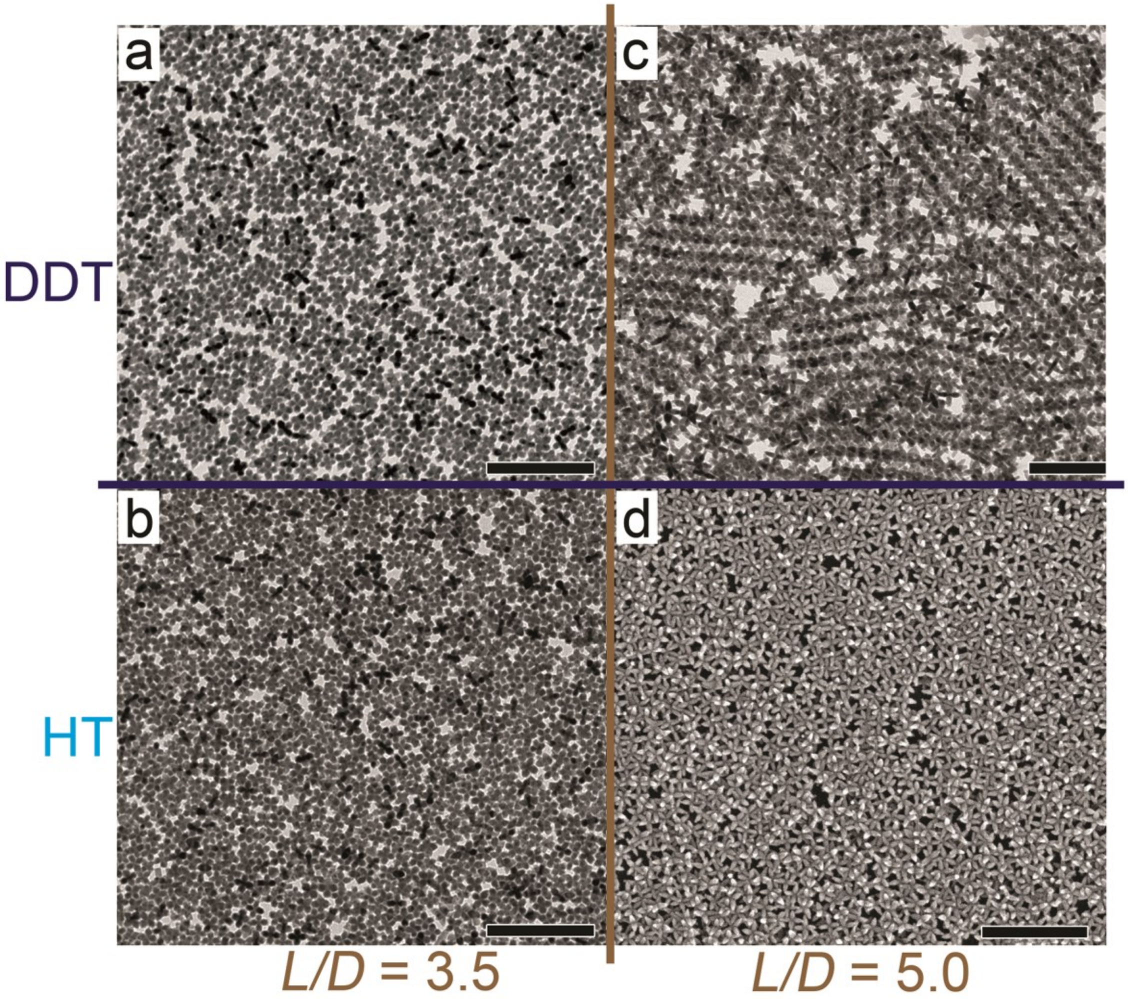

Supplementary Figure 32. Planar superlattices formed by (a, c) DDT- and (b, d) HT-exchanged octapods with L/D of 3.5 (TEM images on a and b panels) and 5.0 (SEM images on c and d panels) at 5 nM of octapods. Scale bars: 200 nm. Octapods with a low L/D do not show variations on their self-assembled configuration, although a closer packing appears to be induced by the shorter thiol ligand (HT). In the case of longer pods, a strong aggregation is observed from samples prepared with the longer thiol ligand (DDT), while the planar superlattices produced with the shorter thiol appear to break the interlocked-chains observed from as-synthesized octapods; only very few short chains are observed in the membranes.

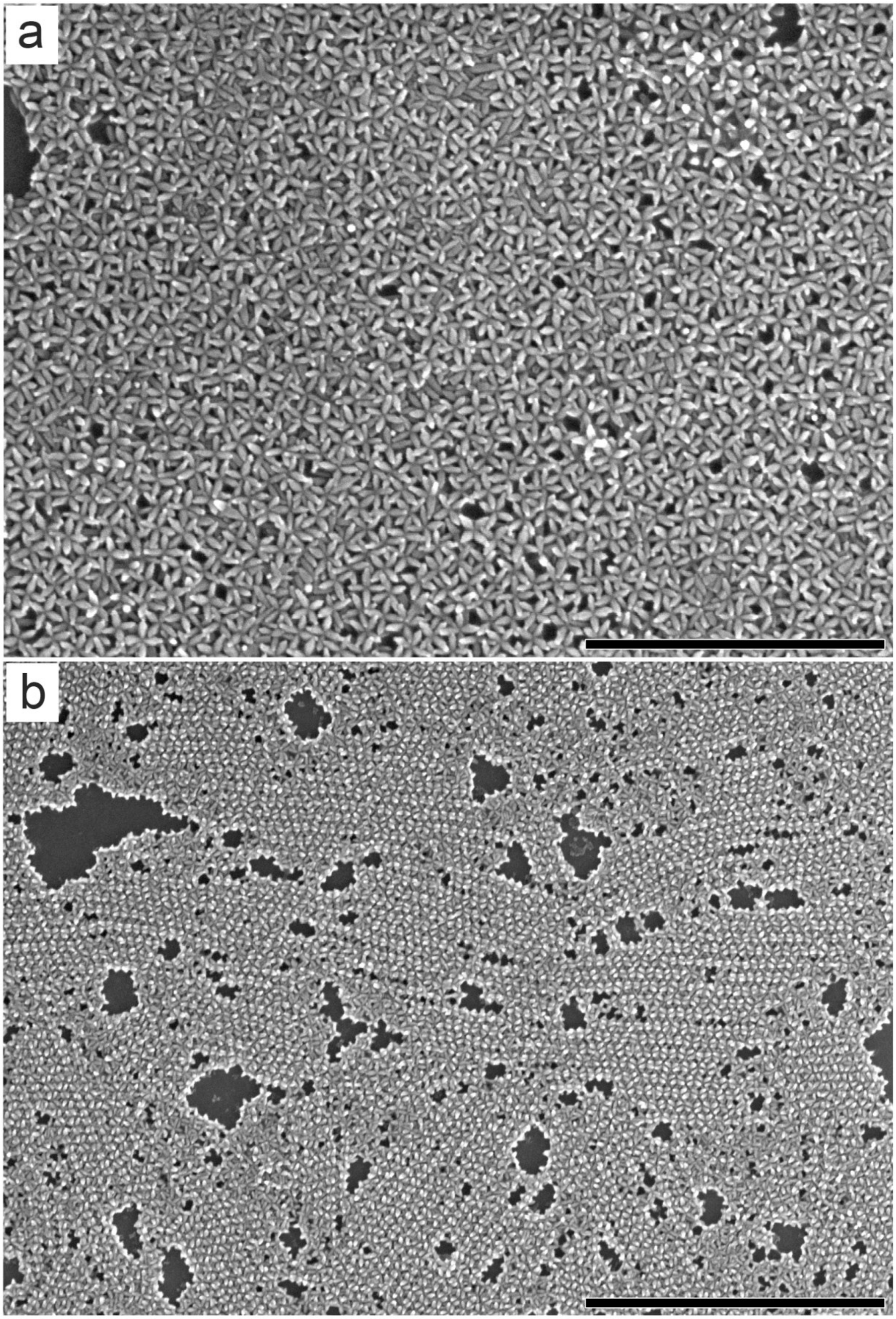

Supplementary Figure 33. Strikingly different configuration of octapods on planar superlattices formed from (a) HT- and (b) DDT-ligand exchanged particles when working at octapod densities of 1 nM and 0.5 nM, respectively. Scale bars: (a) 500 nm; (b) 1 μm.

Supplementary Note 9: Study of octapod surfaces via high-resolution Nuclear Magnetic Resonance (NMR).

HR-NMR analyses were performed on the pure phosphorus-based ligands used in the synthesis of octapods to verify the selectivity of the NMR methods. Toluene-d8, chloroform-d ($CDCl_3$), and tetrahydrofuran-d (THF-d8) were tested to confirm the complete dissolution of the ligands and find a common solvent for all the molecules. All the studied ligands (ODPA, HPA, TOP, and TOPO), with the exception of ODPA, were soluble in both $CDCl_3$ and in toluene-d8, and their proton NMR (^1H NMR) spectra satisfied the criteria of selectivity for at least one resonance. In THF-d8, instead, all the ligands showed a high solubility, but an inefficient selectivity. For this reason, we then performed phosphorus-31 NMR (^{31}P-NMR) measurements on the ligands, from which a high selectivity was achieved for THF-d8 solutions. We note, however, that in the analysis the resonances obtained for HPA and ODPA are extremely similar. Supplementary Figure 34 collects all the acquired ^{31}P-NMR spectra from the pure ligands and it includes the results obtained for octapods with two different aspect ratios, L/D of 3.0 and 6.0, respectively.

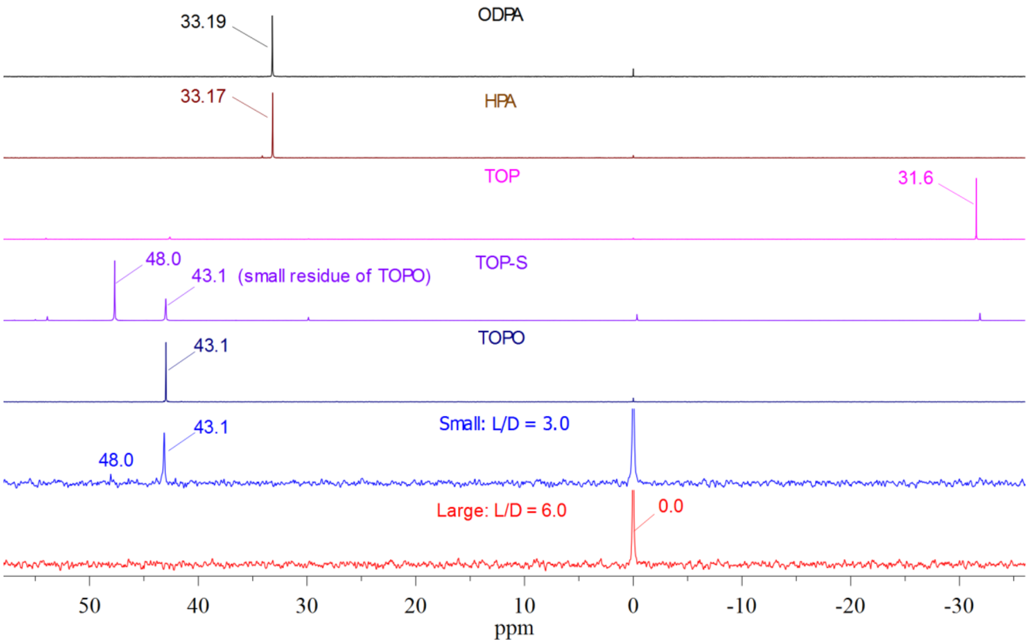

Supplementary Figure 34. ^{31}P-NMR (^1H-decoupled) NMR spectra of the ligands used in the synthesis of octapods and octapods with an L/D of 3.0 and 6.0, respectively. All the ligand solutions and the particle suspensions were prepared in THF-d8.

The spectrum of the octapods with an L/D of 3.0 (recorded from a solution at 250 nM) shows the TOPO peak at ca. 43.1 ppm and, at lowest field, a nearby peak at 48.0 ppm, which was

unambiguously identified with the TOP-S, that derives from synthesis procedure. Sensitivity limits of the ^{31}P-NMR technique likely prevent the detectability of the other ligands, due also to their limited presence in the ligand shell and the relatively low concentration of the particles in the studied suspensions. In the case of the octapods with a larger aspect ratio, the poor solubility of the particles at the relatively high concentration (ca. 250 nM) needed for the ^{31}P NMR studies, significantly affected the recorded spectrum: no signal of the ligands was detected.

Supplementary References

1. Saldanha P. L., Brescia R., Prato M., Li H., Povia M., Manna L., *et al.* Generalized One-Pot Synthesis of Copper Sulfide, Selenide-Sulfide, and Telluride-Sulfide Nanoparticles. *Chem. Mater.* **26**, 1442-1449 (2014).
2. Deka S., Miszta K., Dorfs D., Genovese A., Bertoni G., Manna L. Octapod-Shaped Colloidal Nanocrystals of Cadmium Chalcogenides via "One-Pot" Cation Exchange and Seeded Growth. *Nano Lett.* **10**, 3770-3776 (2010).
3. Kim M. R., Miszta K., Povia M., Brescia R., Christodoulou S., Prato M., *et al.* Influence of chloride ions on the synthesis of colloidal branched CdSe/CdS nanocrystals by seeded growth. *ACS Nano.* **6**, 11088-11096 (2012).
4. Brescia R., Miszta K., Dorfs D., Manna L., Bertoni G. Birth and Growth of Octapod-Shaped Colloidal Nanocrystals Studied by Electron Tomography. *J. Phys. Chem. C.* **115**, 20128-20133 (2011).
5. Xiao Q., Xiao C. Surface-defect-states photoluminescence in CdS nanocrystals prepared by one-step aqueous synthesis method. *App. Surf. Sci.* **255**, 7111-7114 (2009).
6. Dong A., Chen J., Vora P. M., Kikkawa J. M., Murray C. B. Binary nanocrystal superlattice membranes self-assembled at the liquid–air interface. *Nature.* **466**, 474-477 (2010).
7. Barnard A. S., Xu H. First Principles and Thermodynamic Modeling of CdS Surfaces and Nanorods. *J. Phys. Chem. C.* **111**, 18112-18117 (2007).
8. Fessenden R. J., Fessenden J. S. *Organic Chemistry*, 5th Edition edn. Brook-Cole: Pacific Groove, CA, 1994.
9. Smecca E., Motta A., Fragalà M. E., Aleeva Y., Condorelli G. G. Spectroscopic and Theoretical Study of the Grafting Modes of Phosphonic Acids on ZnO Nanorods. *J. Phys. Chem. C.* **117**, 5364-5372 (2013).
10. Hambrock J., Birkner A., Fischer R. A. Synthesis of CdSe nanoparticles using various organometallic cadmium precursors. *J. Mater. Chem.* **11**, 3197-3201 (2001).
11. Ahmed S., Ryan K. M. Centimetre scale assembly of vertically aligned and close packed semiconductor nanorods from solution. *Chem. Commun.* **0**, 6421-6423 (2009).
12. O'Sullivan C., Crilly S., Laffir F. R., Singh A., Magner E., Ryan K. M. Protein immobilisation on perpendicularly aligned gold tipped nanorod assemblies. *Chem. Commun.* **47**, 2655-2657 (2011).
13. Ahmed S., Ryan K. M. Centimetre scale assembly of vertically aligned and close packed semiconductor nanorods from solution. *Chem. Commun.* 6421-6423 (2009).
14. Vega C., Lago S. A fast algorithm to evaluate the shortest distance between rods. *Computers & Chemistry.* **18**, 55-59 (1994).
