# Supplementary material for: Understanding and tailoring ligand interactions in the self-assembly of branched colloidal nanocrystals into planar superlattices
Source: Nat Commun. 2018 Mar 20;9:1141. doi: 10.1038/s41467-018-03550-z (PMC5861251; doi:10.1038/s41467-018-03550-z)
Supplement: Supplementary file 3 — Description of Additional Supplementary Information(PDF 71 kb) [file 41467_2018_3550_MOESM3_ESM.pdf]

Description of Additional Supplementary Files

File Name: Supplementary Movie 1
Description: Isosurface display of volume reconstruction of two octapods with L/D of 3.0.

File Name: Supplementary Movie 2
Description: Isosurface display of volume reconstruction of two octapods with L/D of 6.0.
